# Publisher Correction: Soil contamination in nearby natural areas mirrors that in urban greenspaces worldwide

**DOI:** 10.1038/s41467-023-37920-z

**Published:** 2023-04-26

**Authors:** Yu-Rong Liu, Marcel G. A. van der Heijden, Judith Riedo, Carlos Sanz-Lazaro, David J. Eldridge, Felipe Bastida, Eduardo Moreno-Jiménez, Xin-Quan Zhou, Hang-Wei Hu, Ji-Zheng He, José L. Moreno, Sebastian Abades, Fernando Alfaro, Adebola R. Bamigboye, Miguel Berdugo, José L. Blanco-Pastor, Asunción de los Ríos, Jorge Duran, Tine Grebenc, Javier G. Illán, Thulani P. Makhalanyane, Marco A. Molina-Montenegro, Tina U. Nahberger, Gabriel F. Peñaloza-Bojacá, César Plaza, Ana Rey, Alexandra Rodríguez, Christina Siebe, Alberto L. Teixido, Nuria Casado-Coy, Pankaj Trivedi, Cristian Torres-Díaz, Jay Prakash Verma, Arpan Mukherjee, Xiao-Min Zeng, Ling Wang, Jianyong Wang, Eli Zaady, Xiaobing Zhou, Qiaoyun Huang, Wenfeng Tan, Yong-Guan Zhu, Matthias C. Rillig, Manuel Delgado-Baquerizo

**Affiliations:** 1 State Key Laboratory of Agricultural Microbiology, Huazhong Agricultural University, Wuhan, 430070 China; 2 College of Resources and Environment, Huazhong Agricultural University, Wuhan, 430070 China; 3 Plant-Soil Interactions, Agroscope, Zürich, Switzerland; 4 Department of Plant and Microbial Biology, University of Zürich, Zürich, Switzerland; 5 Multidisciplinary Institute for Environmental Studies (MIES), University of Alicante, P.O. Box 99, Alicante, E-03080 Spain; 6 Department of Ecology, University of Alicante, PO Box 99, Alicante, E-03080 Spain; 7 Centre for Ecosystem Science, School of Biological, Earth and Environmental Sciences, University of New South Wales, Sydney, NSW 2052 Australia; 8 CEBAS-CSIC. Department of Soil and Water Conservation. Campus Universitario de Espinardo, 30100 Murcia, Spain; 9 Department of Agricultural and Food Chemistry, Faculty of Sciences, Universidad Autónoma de Madrid, 28049 Madrid, Spain; 10 Institute of Biology, Freie Universität Berlin, Berlin, 14195 Germany; 11 Berlin-Brandenburg Institute of Advanced Biodiversity Research (BBIB), Berlin, 14195 Germany; 12 Faculty of Science, The University of Melbourne, Parkville, 3010 VIC Australia; 13 GEMA Center for Genomics, Ecology & Environment, Universidad Mayor, Santiago, Chile; 14 Instituto de Ecología y Biodiversidad (IEB), Santiago, 7800003 CP Chile; 15 Natural History Museum (Botany Unit), Obafemi Awolowo University, Ile-Ife, Nigeria; 16 Departamento de Biodiversidad, Ecología y Evolución, Facultad de Biología, Universidad Complutense de Madrid, C/Jose Antonio Novais 12, Madrid, 28040 Spain; 17 INRAE, UR4 (URP3F), Centre Nouvelle-Aquitaine-Poitiers, Lusignan, 86600 France; 18 Museo Nacional de Ciencias Naturales, Consejo Superior de Investigaciones Científicas, Serrano 115 bis, 28006 Madrid, Spain; 19 Misión Biológica de Galicia, Consejo Superior de Investigaciones Científicas, Pontevedra, Spain; 20 Centre for Functional Ecology, Department of Life Sciences, University of Coimbra, 3000-456 Coimbra, Portugal; 21 Department of Forest Physiology and Genetics, Slovenian Forestry Institute, Ljubljana, Slovenia; 22 Department of Entomology, Washington State University, Pullman, WA 99164 USA USA; 23 Department of Biochemistry, Genetics and Microbiology, DSI/NRF SARChI Chair in Marine Microbiomics, University of Pretoria, Pretoria, 0028 South Africa; 24 Centre for Integrative Ecology, ICB, Universidad de Talca, Talca, Chile; 25 CEAZA, Universidad Católica del Norte, Coquimbo, Chile; 26 Laboratório de Sistemática Vegetal, Departamento de Botânica, Instituto de Ciências Biológicas, Universidade Federal de Minas Gerais, Av. Antônio Carlos, 6627, Pampulha, Belo Horizonte, 31270-901 MG Brazil; 27 Instituto de Ciencias Agrarias, Consejo Superior de Investigaciones Científicas, Serrano 115 bis, 28006 Madrid, Spain; 28 Instituto de Geología, Universidad Nacional Autónoma de México, Ciudad Universitaria, México D.F, 04510 CP México; 29 Departamento de Botânica e Ecologia, Instituto de Biociências, Universidade Federal de Mato Grosso, Av. Fernando Corrêa, 2367, Boa Esperança, Cuiabá, 78060-900 MT Brazil; 30 Microbiome Network and Department of Agricultural Biology, Colorado State University, Fort Collins, 80523 CO USA; 31 Grupo de Biodiversidad y Cambio Global (BCG), Departamento de Ciencias. Básicas, Universidad del Bío-Bío, Campus Fernando May, Chillán, Chile; 32 Plant-Microbes Interaction Lab, Institute of Environment and Sustainable Development, Banaras Hindu University, Varanasi, 221005 Uttar Pradesh India; 33 Institute of Grassland Science/School of Life Science, Northeast Normal University, and Key Laboratory of Vegetation Ecology, Ministry of Education, Changchun, 130024 Jilin, China; 34 Department of Natural Resources, Agricultural Research Organization, Institute of Plant Sciences, Gilat Research Center, Negev, 8531100 Israel; 35 State Key Laboratory of Desert and Oasis Ecology, Xinjiang Institute of Ecology and Geography, Chinese Academy of Sciences, Urumqi, China; 36 State Environmental Protection Key Laboratory of Soil Health and Green Remediation, Wuhan, 430000 China; 37 State Key Laboratory of Urban and Regional Ecology, Research Center for Eco-Environmental Sciences, Chinese Academy of Sciences, Beijing, 100085 China; 38 Laboratorio de Biodiversidad y Funcionamiento Ecosistémico. Instituto de Recursos Naturales y Agrobiología de Sevilla (IRNAS), CSIC, Av. Reina Mercedes 10, Sevilla, E-41012 Spain; 39 Unidad Asociada CSIC-UPO (BioFun)., Universidad Pablo de Olavide, Sevilla, 41013 Spain

**Keywords:** Environmental sciences, Ecology

Correction to: *Nature Communications* 10.1038/s41467-023-37428-6, published online 27 March 2023

In the version of this article originally published, the current affiliation 25, “CEAZA, Universidad Católica del Norte, Coquimbo, Chile,” initially appeared as the last affiliation, offsetting all author footnotes from 25-39. The affiliation order has been restored in the article.

