# Supplementary material for: Soil contamination in nearby natural areas mirrors that in urban greenspaces worldwide
Source: Nat Commun. 2023 Mar 27;14:1706. doi: 10.1038/s41467-023-37428-6 (PMC10042830; doi:10.1038/s41467-023-37428-6)
Supplement: Supplementary file 1 — Supplementary Information [file 41467_2023_37428_MOESM1_ESM.pdf]

Supplementary Information

Soil contamination in nearby natural areas mirrors that in urban greenspaces worldwide

Yu-Rong Liu[1,2*], Marcel G.A. van der Heijden[3,4], Judith Riedo[4], Carlos Sanz-Lazaro[5,6], David J. Eldridge[7], Felipe Bastida[8], Eduardo Moreno-Jiménez[9,10,11], Xin-Quan Zhou[2], Hang-Wei Hu[12], Ji-Zheng He[12], José L. Moreno[8], Sebastian Abades[13], Fernando Alfaro[13,14], Adebola R. Bamigboye[15], Miguel Berdugo[16], José L. Blanco-Pastor[17], Asunción de los Ríos[18], Jorge Duran[19,20], Tine Grebenc[21], Javier G. Illán[22], Thulani P. Makhalanyane[23], Marco A. Molina-Montenegro[24], Tina U. Nahberger[21] Gabriel F. Peñaloza-Bojacá[25], César Plaza[26], Ana Rey[19], Alexandra Rodríguez[19,20], Christina Siebe[27], Alberto L. Teixido[28], Nuria Casado-Coy[5], Pankaj Trivedi[29], Cristian Torres-Díaz[30], Jay Prakash Verma[31], Arpan Mukherjee[31], Xiao-Min Zeng[2], Ling Wang[32], Jianyong Wang[32], Eli Zaady[33], Xiaobing Zhou[34], Qiaoyun Huang[1, 2], Wenfen Tan[2,35], Yong-Guan Zhu[36], Matthias C. Rillig[10,11], Manuel Delgado-Baquerizo[37,38*]

[1]State Key Laboratory of Agricultural Microbiology, Huazhong Agricultural University, 430070 Wuhan, China

[2]College of Resources and Environment, Huazhong Agricultural University, 430070 Wuhan, China

[3]Plant-Soil Interactions, Institute for Sustainability Sciences, Agroscope, Zürich, 8046, Switzerland

[4]Institute of Evolutionary Biology and Environmental Studies, University of Zürich, Zürich, 8057, Switzerland

[5]Multidisciplinary Institute for Environmental Studies (MIES), University of Alicante, P.O. Box 99, E-03080, Alicante, Spain

[6]Department of Ecology, University of Alicante, PO Box 99, E-03080, Alicante, Spain

[7]Centre for Ecosystem Science, School of Biological, Earth and Environmental Sciences, University of New South Wales, Sydney, New South Wales 2052, Australia

[8]CEBAS-CSIC. Department of Soil and Water Conservation. Campus Universitario de Espinardo, 30100, Murcia, Spain

[9]Department of Agricultural and Food Chemistry, Faculty of Sciences, Universidad Autónoma de Madrid, 28049, Madrid, Spain

[10]Institute of Biology, Freie Universität Berlin, Berlin, 14195 Germany

[11]Berlin-Brandenburg Institute of Advanced Biodiversity Research (BBIB), Berlin, 14195 Germany

[12]Faculty of Science, The University of Melbourne, Parkville 3010, Victoria, Australia

[13]GEMA Center for Genomics, Ecology & Environment, Universidad Mayor, Santiago, Chile

[14] Instituto de Ecología y Biodiversidad (IEB), CP 7800003, Santiago, Chile

[15]Natural History Museum (Botany Unit), Obafemi Awolowo University, Ile-Ife, Nigeria

[16]Institute of Integrative Biology, Department of Environment Systems Science, ETH Zürich, 8092 Zürich, Switzerland

[17]INRAE, UR4 (URP3F), Centre Nouvelle-Aquitaine-Poitiers, 86600 Lusignan, France

[18]Museo Nacional de Ciencias Naturales, Consejo Superior de Investigaciones Científicas, Serrano 115 bis, 28006 Madrid, Spain

[19]Misión Biológica de Galicia, Consejo Superior de Investigaciones Científicas, Pontevedra, Spain.

[20]Centre for Functional Ecology, Department of Life Sciences, University of Coimbra, 3000-456 Coimbra, Portugal

[21]Department of Forest Physiology and Genetics, Slovenian Forestry Institute. Ljubljana, Slovenia

[22]Department of Entomology, Washington State University, Pullman, WA. 99164 USA

[23]Centre for Microbial Ecology and Genomics, Department of Biochemistry, Genetics and Microbiology, DSI/NRF SARChI Chair in Marine Microbiomics, University of Pretoria, Pretoria, South Africa, 0028

[24]Laboratorio de Ecología Integrativa, Instituto de Ciencias Biológicas, Universidad de Talca, Talca, Chile, CEAZA, Universidad Católica del Norte, Coquimbo, Chile

[25]Laboratório de Sistemática Vegetal, Departamento de Botânica, Instituto de Ciências Biológicas, Universidade Federal de Minas Gerais, Av. Antônio Carlos, 6627, Pampulha, Belo Horizonte, 31270-901, MG, Brazil

[26]Instituto de Ciencias Agrarias, Consejo Superior de Investigaciones Científicas, Serrano 115 bis, 28006 Madrid, Spain

[27]Instituto de Geología, Universidad Nacional Autónoma de México, Ciudad Universitaria, México D.F. CP 04510, México.

[28]Departamento de Botânnica e Ecologia, Instituto de Biociências, Universidade Federal de Mato Grosso, Av. Fernando Corrêa, 2367, Boa Esperança, Cuiabá, 78060-900, MT, Brazil

[29]Microbiome Network and Department of Agricultural Biology, Colorado State University, Fort Collins, 80523, CO, USA

[30]Grupo de Biodiversidad y Cambio Global (BCG), Departamento de Ciencias. Básicas, Universidad del Bío-Bío, Campus Fernando May, Chillán, Chile

[31]Institute of Environment and Sustainable Development, Banaras Hindu University, Varanasi-221005, Uttar Pradesh, India

[32]Institute of Grassland Science/School of Life Science, Northeast Normal University, and Key Laboratory of Vegetation Ecology, Ministry of Education, Changchun, Jilin 130024, China

[33]Department of Natural Resources, Agricultural Research Organization, Institute of Plant Sciences, Gilat Research Center, Mobile Post Negev, 8531100, Israel

[34]State Key Laboratory of Desert and Oasis Ecology, Xinjiang Institute of Ecology and Geography, Chinese Academy of Sciences, Urumqi, China

[35]State Environmental Protection Key Laboratory of Soil Health and Green Remediation, Wuhan 430000, China

[36]State Key Laboratory of Urban and Regional Ecology, Research Center for Eco-Environmental Sciences, Chinese Academy of Sciences, Beijing 100085, China

[37]Instituto de Recursos Naturales y Agrobiología de Sevilla (IRNAS), CSIC, Av. Reina Mercedes 10, E-41012, Sevilla, Spain.

[38]Departamento de Sistemas Físicos, Químicos y Naturales, Universidad Pablo de Olavide, 41013 Sevilla, Spain

Correspondence to: Yu-Rong Liu; Email: yrliu@mail.hzau.edu.cn;
Manuel Delgado-Baquerizo; Email: m.delgadobaquerizo@gmail.com

This PDF file includes:
Supplementary Method
Supplementary Figures 1 to 18
Supplementary Tables 1 to 9

Supplementary Method: Number of soil contaminants over a threshold

We calculated the number of soil contaminants over four thresholds (e.g., 25%, 50%, 75% and 90%) of maximum contaminant levels acorrding to a similar menthod from previous studies [1,2]. This approach assumes that when a number of soil contaminants (i.e., metal(loid)s, pesticides, microplastics and antibiotic resistance genes) pass high number of contaminanants (e.g., >75%) (i.e., vs. maximum values), this can negatively impact soil mrobial functional traits. Specifically, we first standardized all individual soil contaminants between 0 and 1, and averaged all the standardized levels within each category of contaminant (i.e., metal(loid)s). Then, we calculated the number of the contaminants passing over these four thresholds. Finally, we established relationships between the number of soil contaminants passing different thresholds and the relative abundance of the selected functional genes.

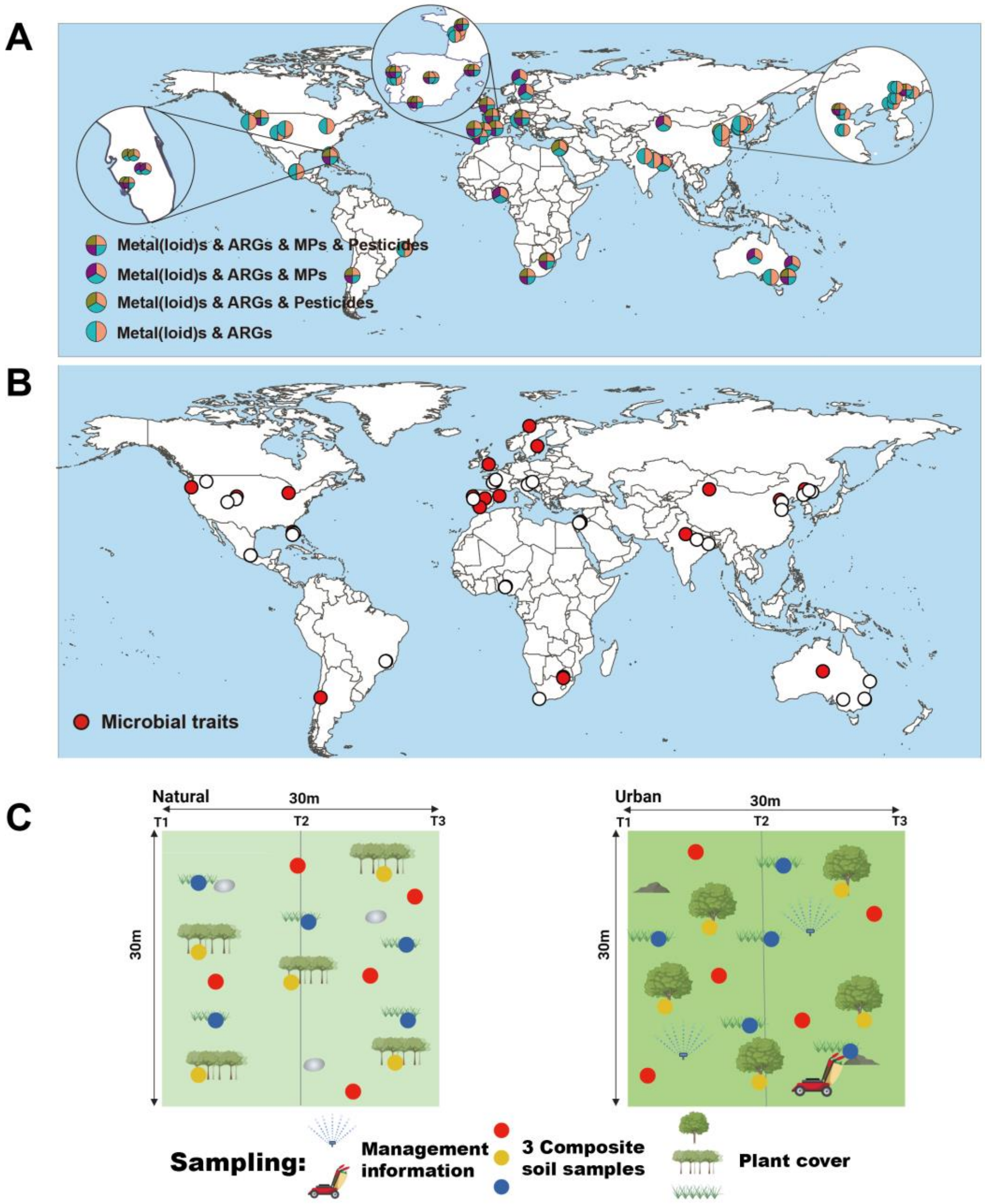

Figure S1. Locations and sampling strategies of the paired natural and urban areas worldwide. Panel A, the sites where soils were used for analyses of contaminants including metal(loid)s, pesticides, microplasitcs (MPs), and antibiotic resistance genes (ARGs) with different colors. Panel B, subsets of the municipalities where soil microbial traits were determined using shotgun sequencing. Red circles represent the locations with microbial analyses (shotgun megetagenomic sequencing), while white ones are the locations without microbial analyses. Panel C, a diagram of sampling design. The number of samples analyzed for each analyses differs due to sample availabilityand resource limitation as explaine in Method. In all cases, these subsets of samples were selected to cover the entire biogeographic range along with contrasting environmental conditions. The maps in figure A and B were generated using ArcGIS 10.2 software, and figure C was drawn in online Biorender (https://www.biorender.com/) using available elements in the platform.

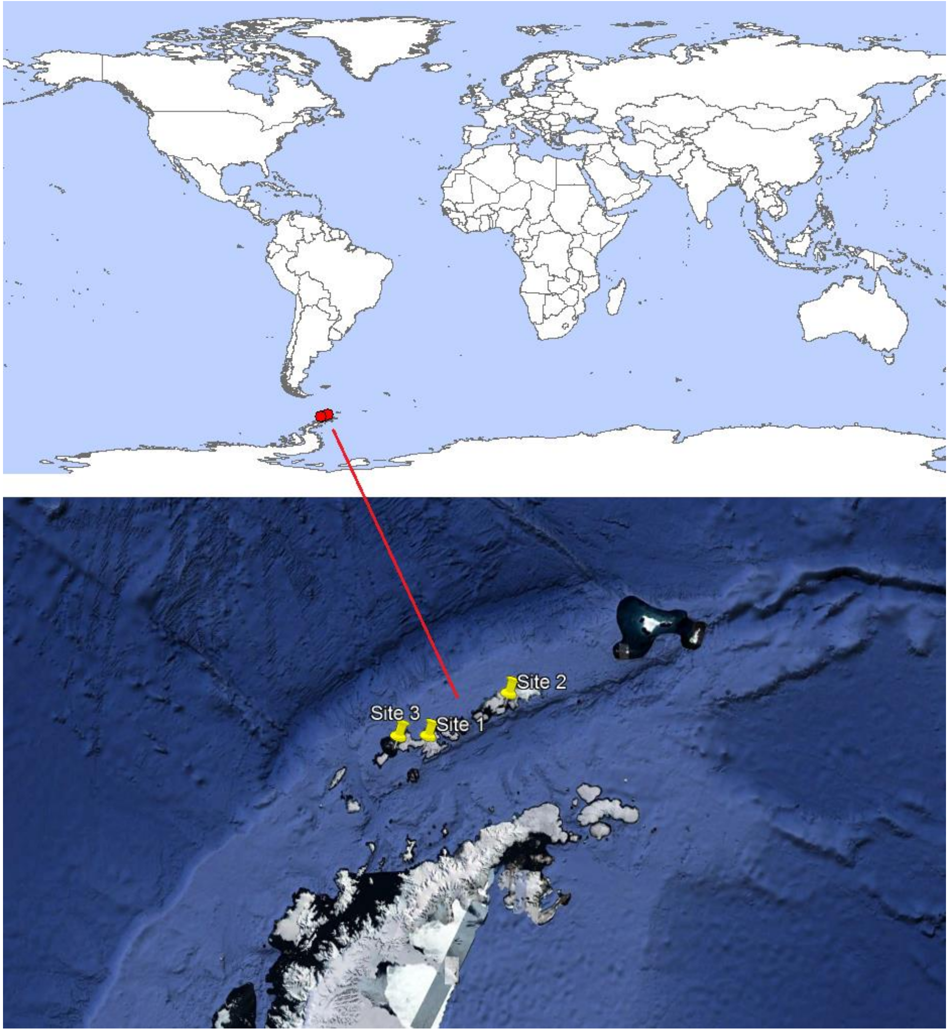

Figure S2. Locations of the three remote ecosystems from maritime Antarctica. The map was generated using ArcGIS 10.2 software.

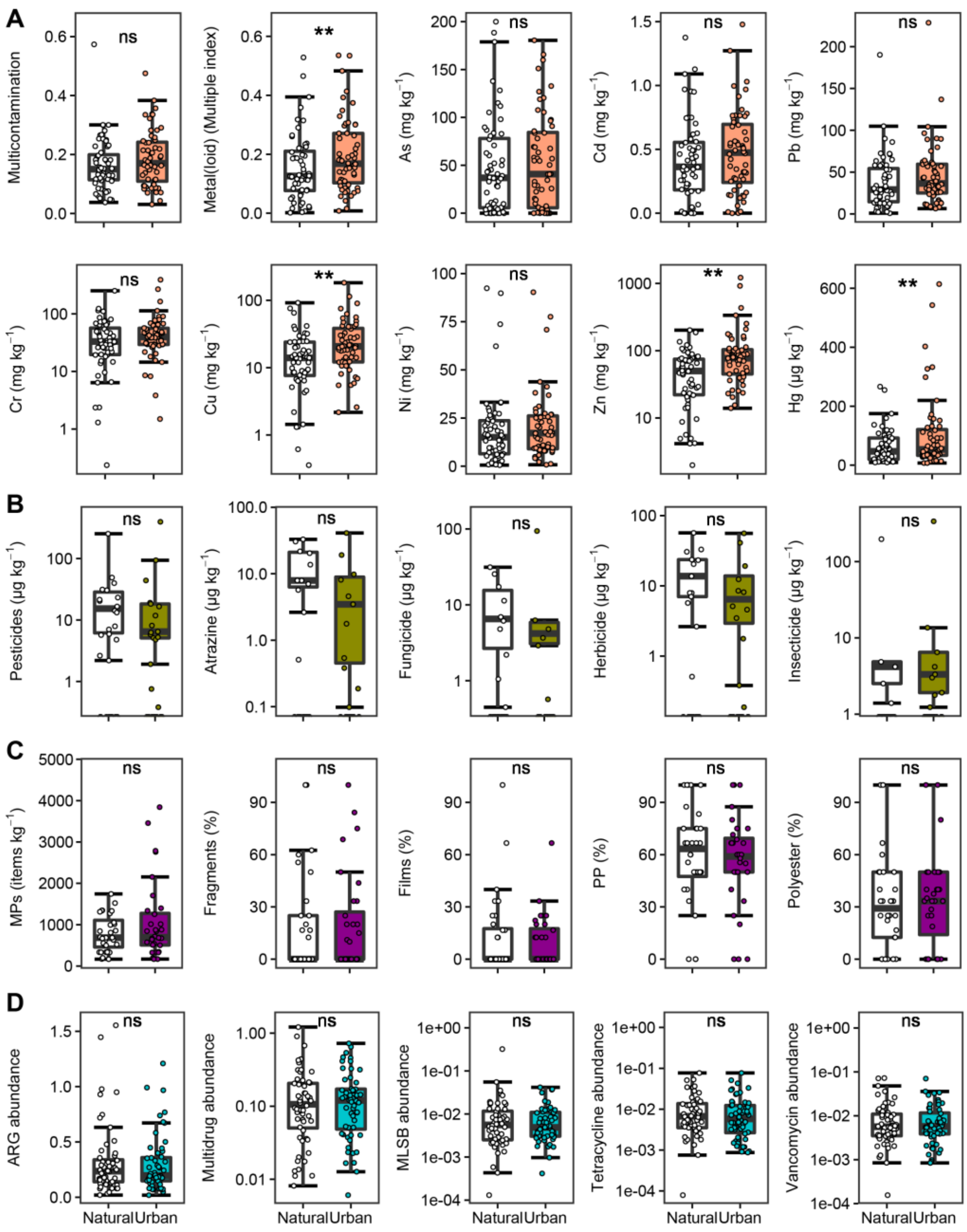

Figure S3. The occurrence of soil contaminants in urban greenspaces and adjacent natural areas. Panel A, soil contents of the individual metal(loid)s (n =112): mercury (Hg), arsenic (As), copper (Cu), cadmium (Cd), lead (Pb), zinc (Zn), nickel (Ni) and chromium (Cr), the average occurrence of multiple metal(loid)s and multi-contamination (see Methods). Panel B, soil contents of all pesticides, one specific herbicide (Atrazine + transformation products), and the assessed fungicides, herbicides and insecticides (n = 54). Panel C, the total content of microplastics (MPs) and percentages of major shapes and polymer types (n = 64). Panel D, proportions of total and selected antibiotic resistance genes (ARGs) (n = 112). Asterisks within each panel indicate significant differences between ecosystems based on nested PERMANOVA analyses using a block design as described in the Methods section. **P* < 0.05; ***P* < 0.01. ns = not significant (see Table S4 for the statistical summary).

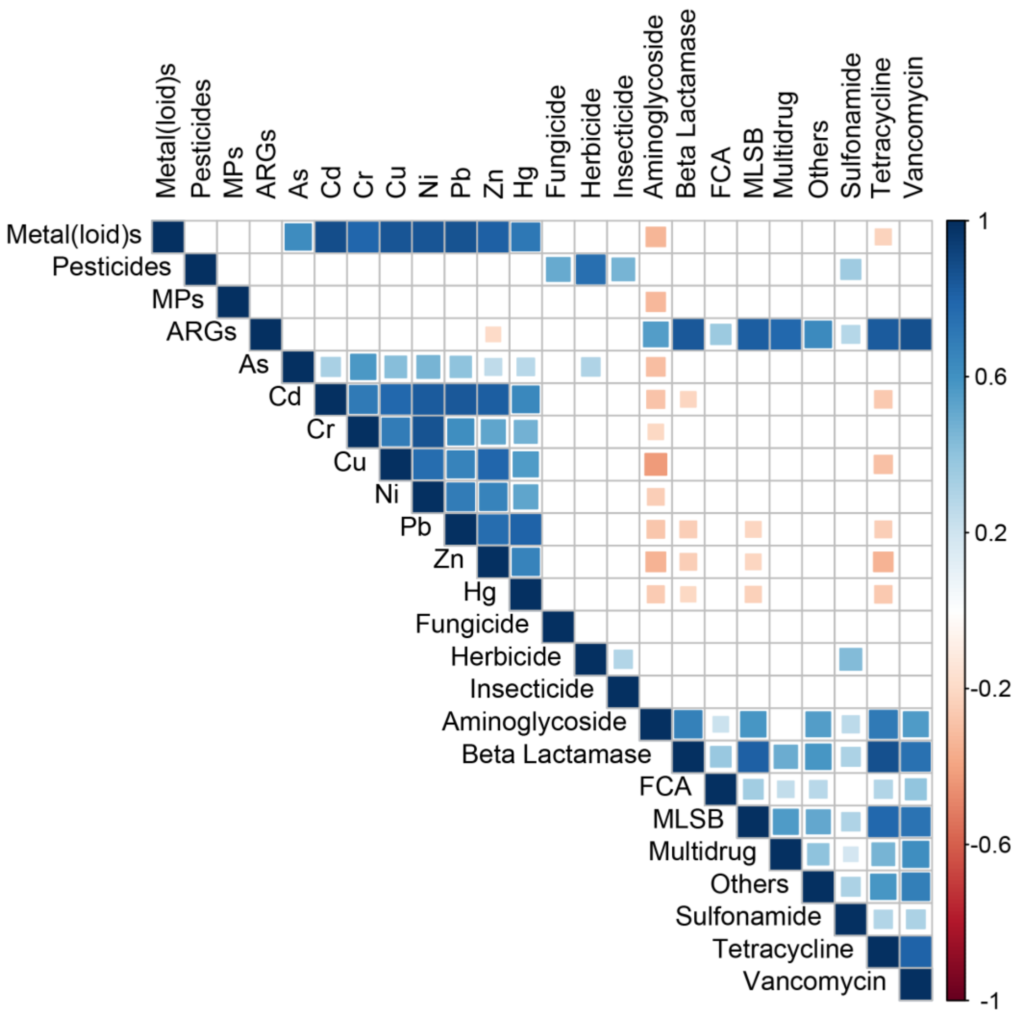

Figure S4. Spearman correlations between contaminants in the soil from urban greenspaces and adjacent natural areas. Metal(loid)s contain mercury (Hg), arsenic (As), copper (Cu), cadmium (Cd), lead (Pb), zinc (Zn), nickel (Ni) and chromium (Cr). The size of the square represents the strength of the relationship when significant. MPs, microplasitcs; ARGs, antibiotic resistance genes; FCA, fluoroquinolone, quinolone, florfenicol, chloramphenicol and amphenicol; MLSB, macrolide-lincosamide-streptogramin B. Statistically non-significant correlations (P > 0.05) are shown in white.

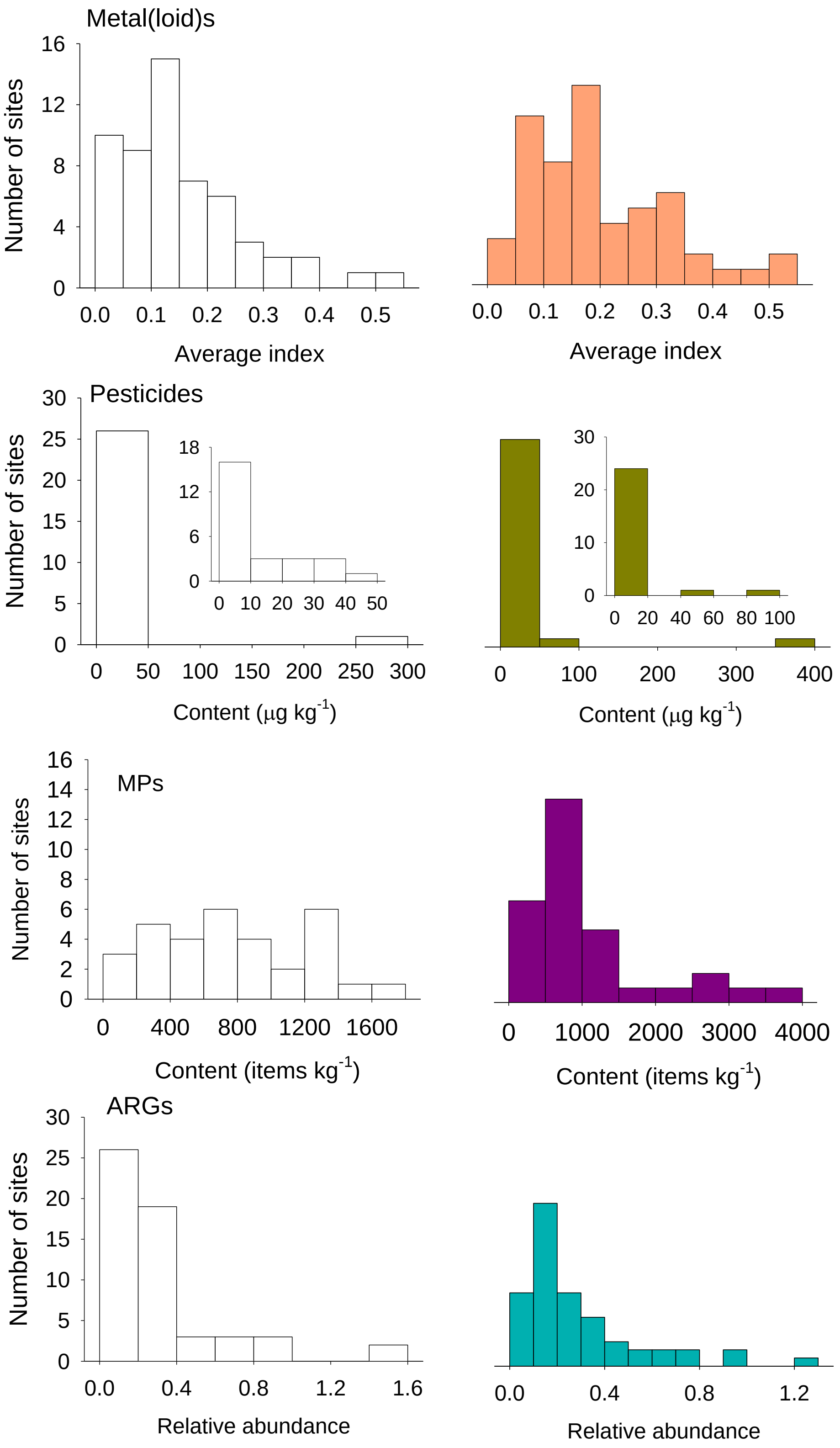

Figure S5. Distributions of metal(loid)s, pesticide residues, microplasitcs (MPs), and antibiotic resistance genes (ARGs) in topsoils of natural areas and urban greenspaces. Metal(loid) indices were calculated by averaging the standardized metal(loid) contents (see Methods). White columns show the distribution of metal(loid)s in natural areas, while color columns show those in urban greenspaces.

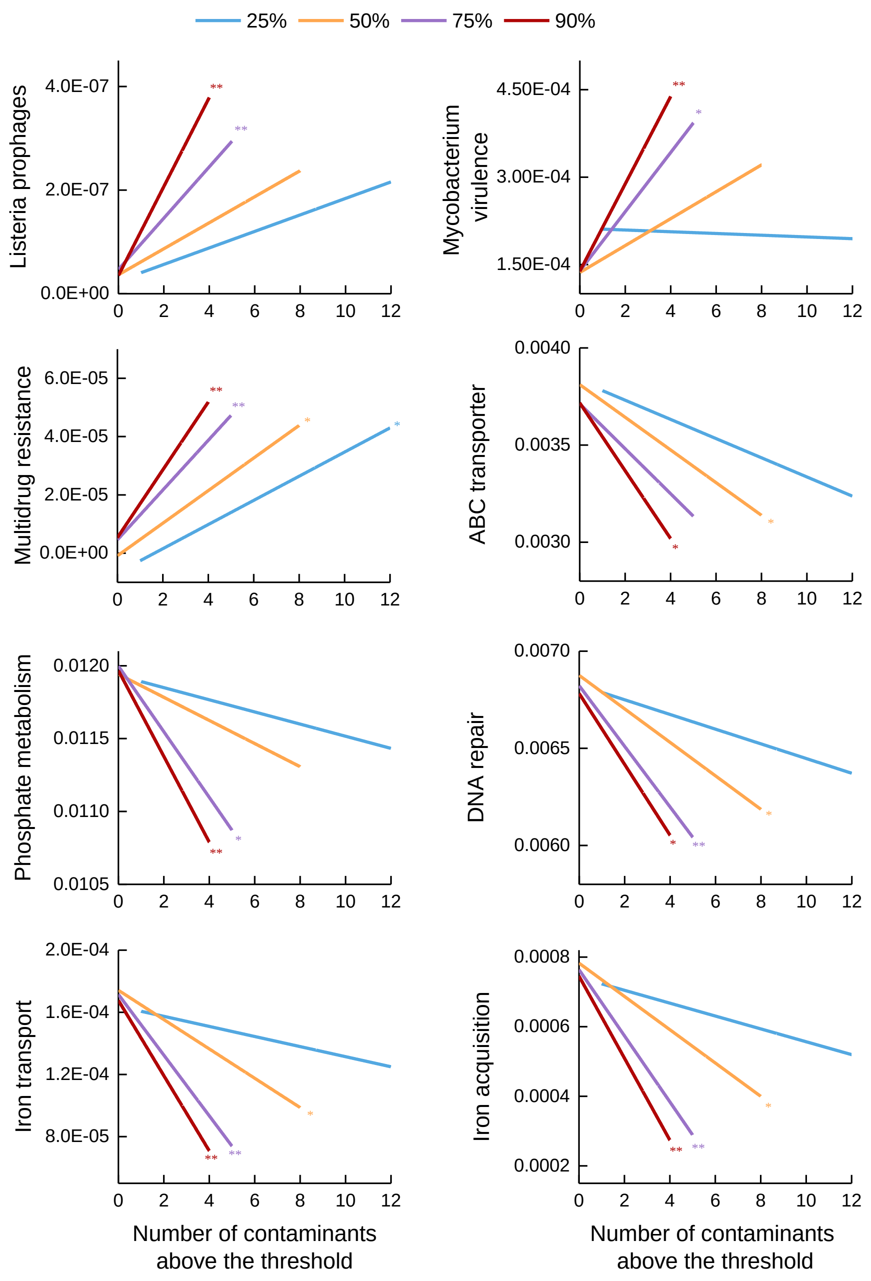

Figure S6. Relationships between the number of contaminants operating above a threshold (measured as % respect to maximum values of contaminants) and relative abundance of the seletced genes. These genes are associated with environmental stress resistance, nutrient cycling, and pathogenesis, and detailed gene category is shownin Supplementary Table 9.

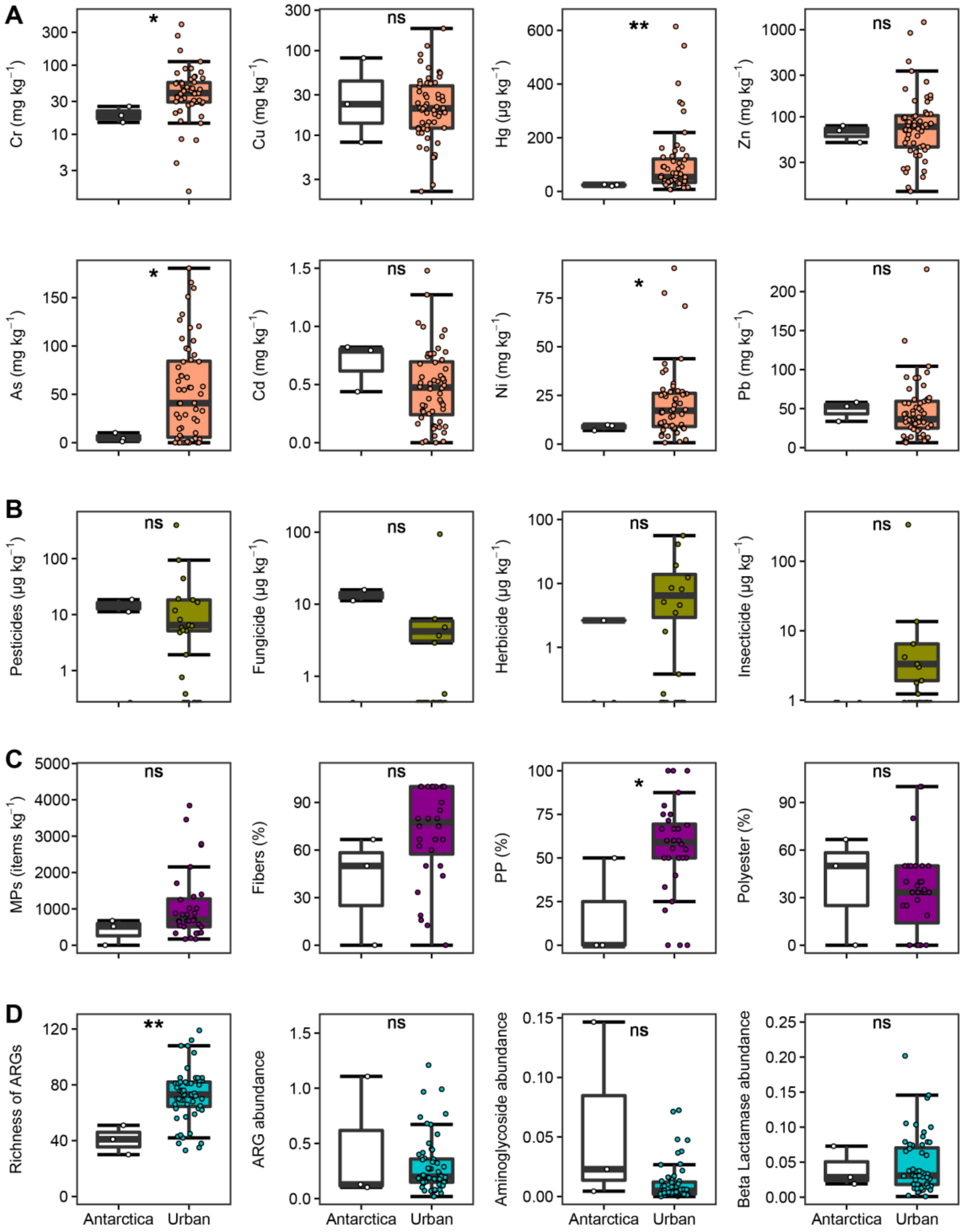

Figure S7. Comparisons of soil contaminants between the Antarctica and urban greenspaces. Asterisks indicate significant differences using ANOVA (detailed statistical test is shown in Figure S8). Panel A, heavy metals; Panel B, pesticides; Panel C, microplastics (MPs), Panel D, antibiotic resistance genes (ARGs). Bootstrap was performed to randomly take 100 times of 3 samples from 56 urban ecosystems for a comparison with 3 samples from Antarctica. *$P < 0.05$; **$P < 0.01$. ns = not significant.

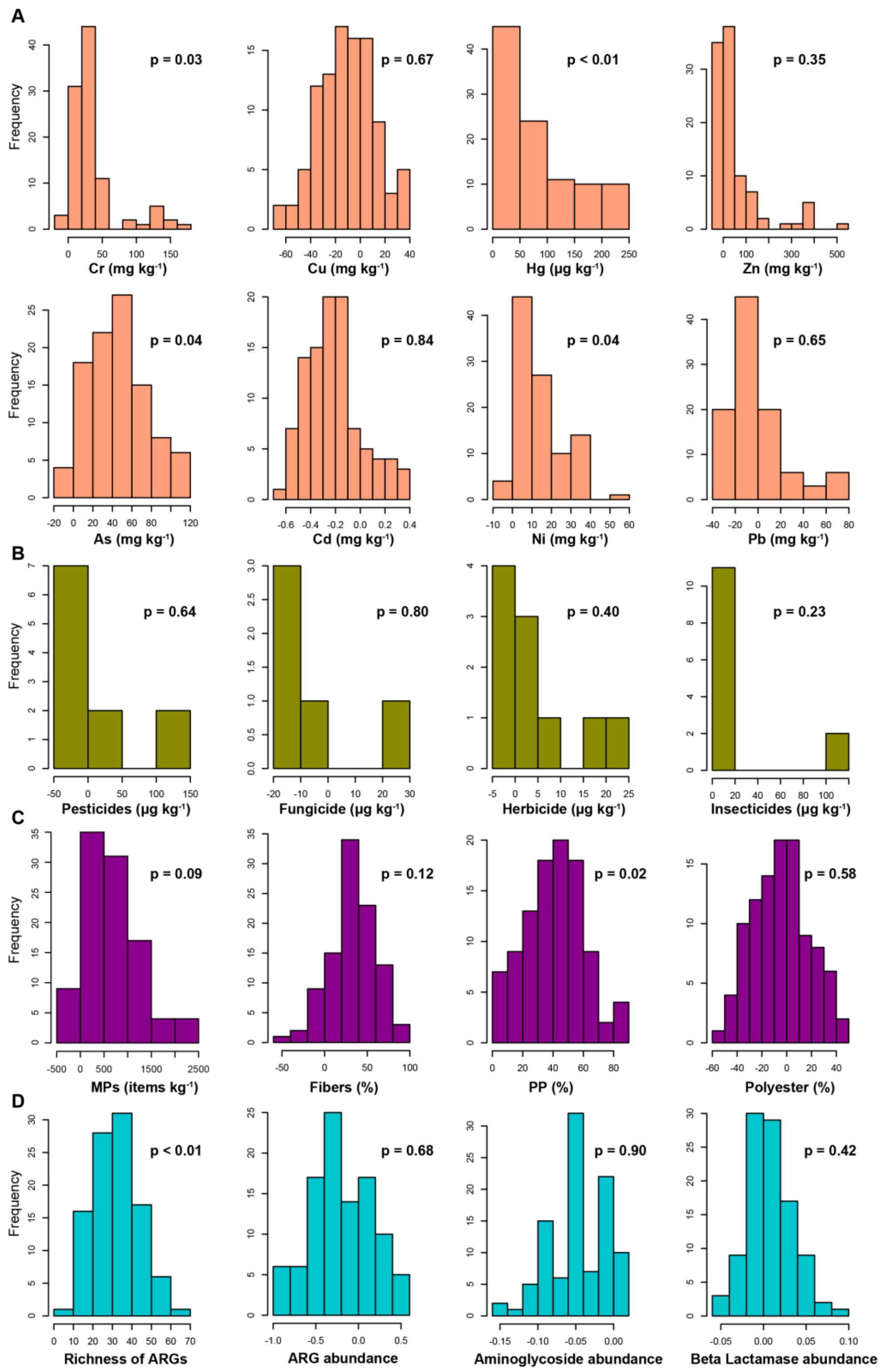

Figure S8. Histograms showing the probability of the differences in soil contaminants between the Antarctica and urban greenspaces (from Figure S7). Panel A, heavy metals; Panel B, pesticides; Panel C, microplastics (MPs), Panel D, antibiotic resistance genes (ARGs). Bootstrap was performed to randomly take 100 times of 3 samples from 56 urban ecosystems for a comparison with 3 samples from Antarctica. The probability (i.e., P-value) ≥ 0.05 represents insignificant differences of soil contaminants between the Antarctica and urban greenspaces.

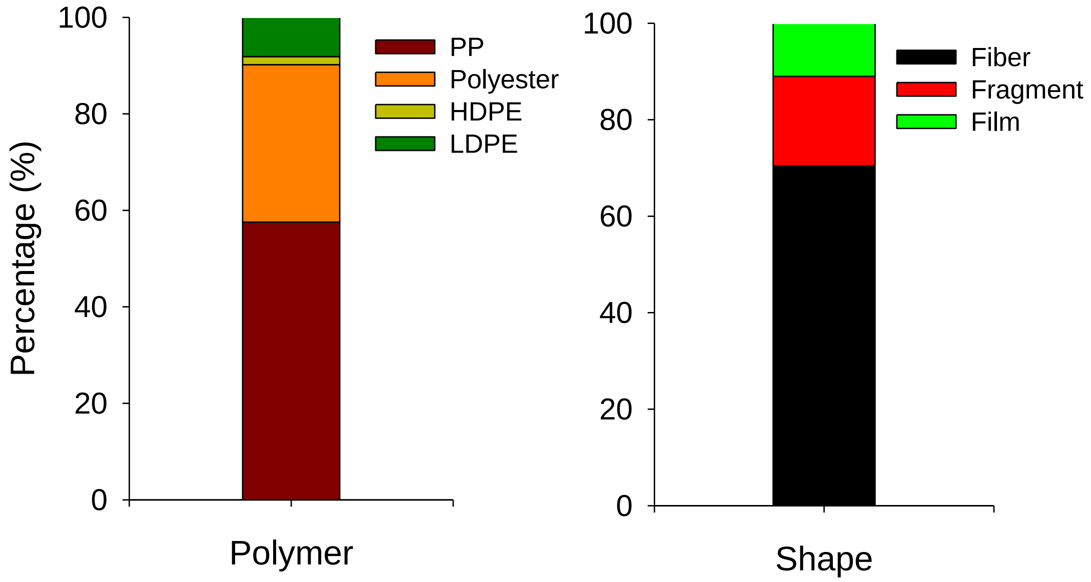

Figure S9. Percentages of polymer type and shape of the microplasitcs (MPs) in the soil of urban greenspaces and adjacent natural areas. PP, polypropylene; HDPE, high-density polyethylene; LDPE, low-density polyethylene.

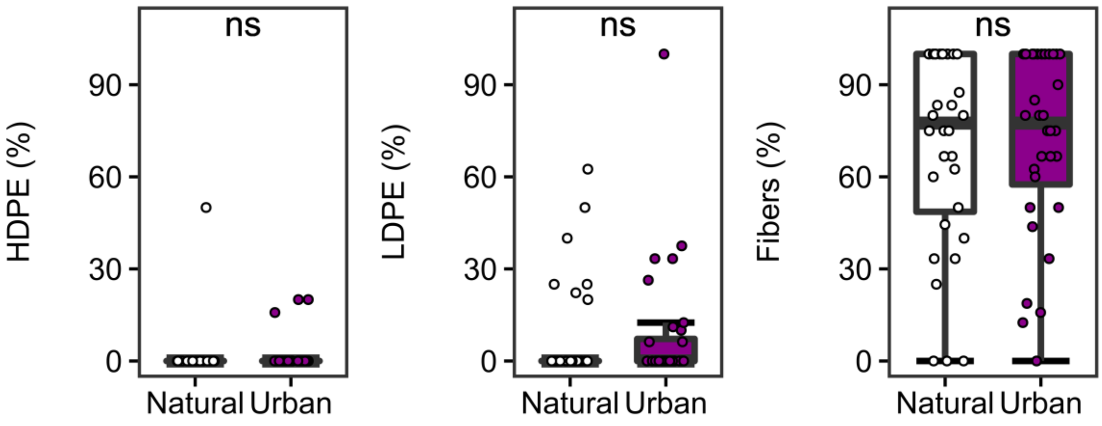

Figure S10. The percentages of other polymer type and shape of soil MPs in urban greenspaces and adjacent natural areas (n = 64). Asterisks within each panel indicate significant differences between ecosystems based on nested PERMANOVA analyses using a block design as described in the Methods section. ns = not significant.

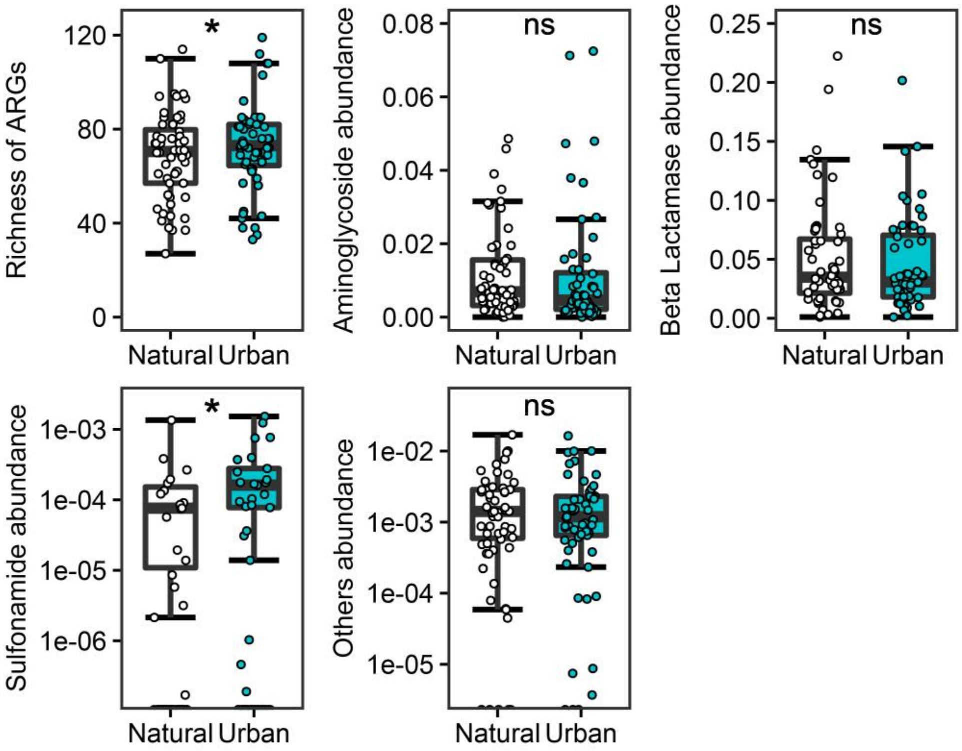

Figure S11. Relative abundances and the richness of remaining antibiotic resistance genes (ARGs) in the soil of natural areas and urban greenspaces (n = 56). Asterisks indicate significant differences in nested PERMANOVA analyses using a block design as described in the Method section. *$P < 0.05$. ns = not significant.

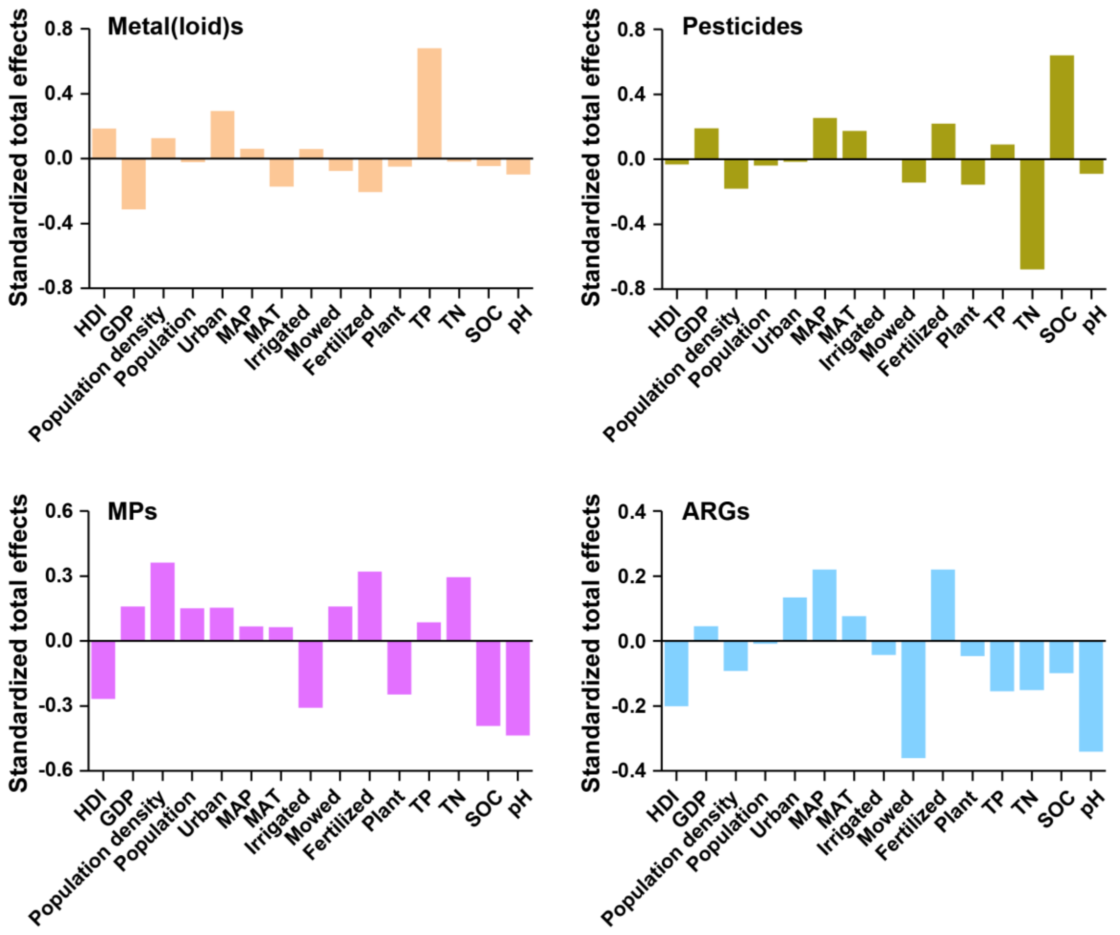

Figure S12. Total standardized effects (direct plus indirect) of different factors on four types of contaminants from the structural equation modelling (Figure 2A in main text). MPs, microplasitcs; ARGs, antibiotic resistance genes; HDI, human development index; GDP, gross domestic product. Urban indicates urban greenspaces, and plant represents plant cover. MAT, mean annual temperature; MAP, mean annual precipitation; SOC, soil organic carbon; TP, total phosphorus; TN, total nitrogen. Urban, urban greenspaces; Plant, plant cover.

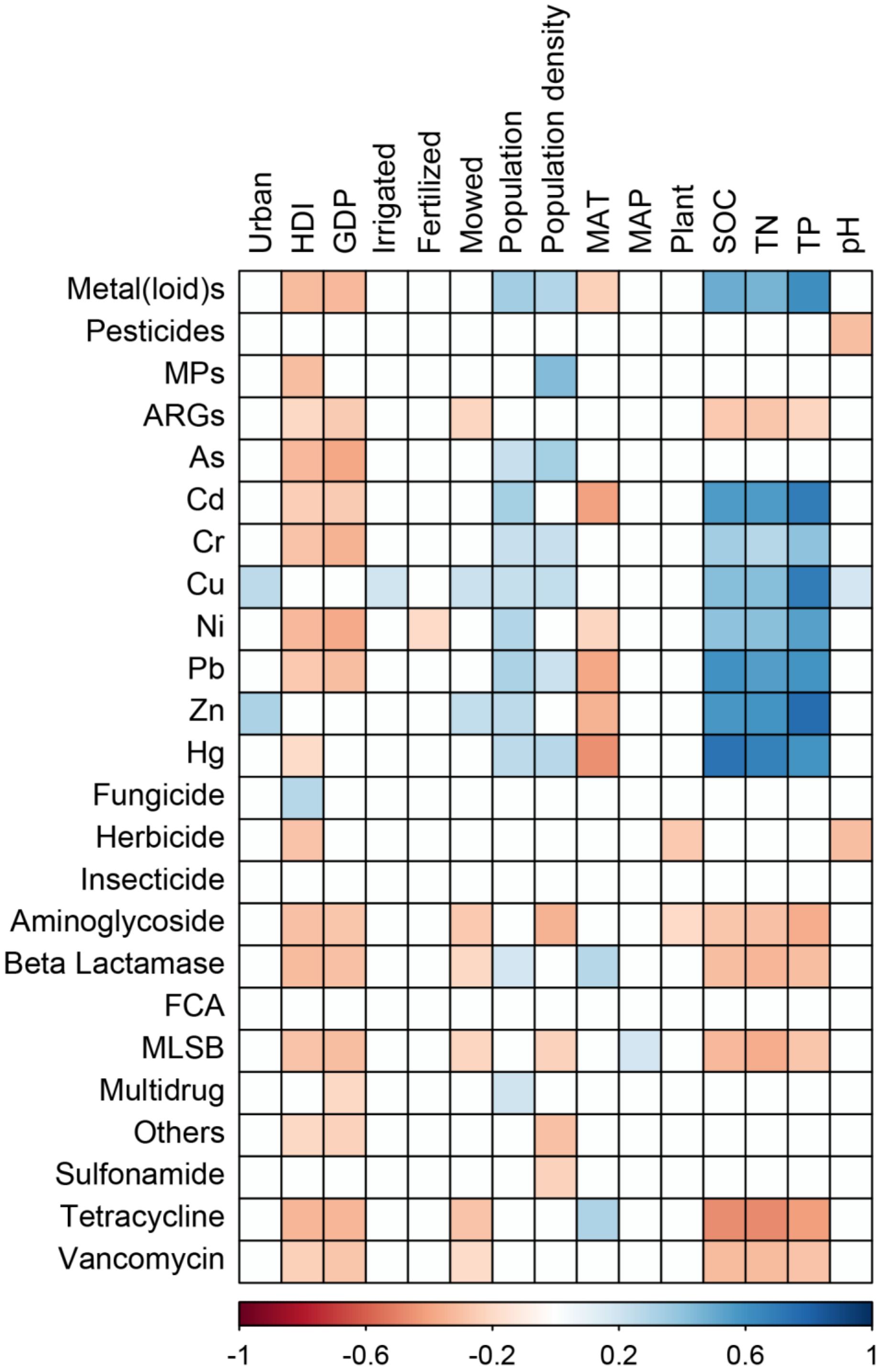

Figure S13. Heatmap showing correlations (Spearman) between soil contaminants and socio-economic and environmental factors. Statistically non-significant correlations ($P > 0.05$) are shown in white. MPs, microplasitcs; ARGs, antibiotic resistance genes; HDI, human development index; GDP, gross domestic product. Urban indicates urban greenspaces, and plant represents plant cover. MAT, mean annual temperature; MAP, mean annual precipitation; SOC, soil organic carbon; TP, total phosphorus; TN, total nitrogen. Urban, urban greenspaces; Plant, plant cover.

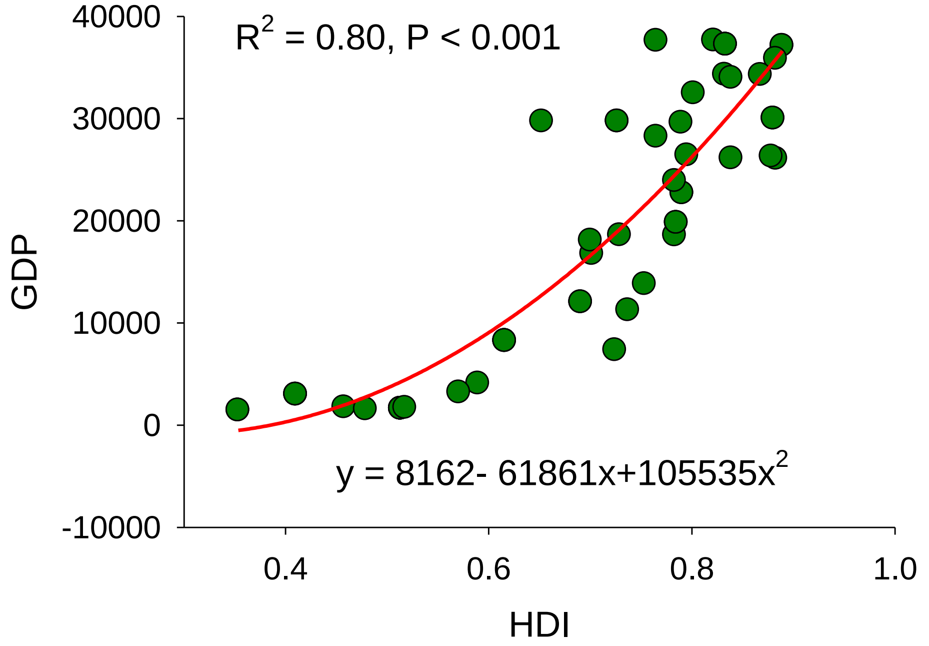

Figure S14. The relationship between human development index (HDI), gross domestic product (GDP).

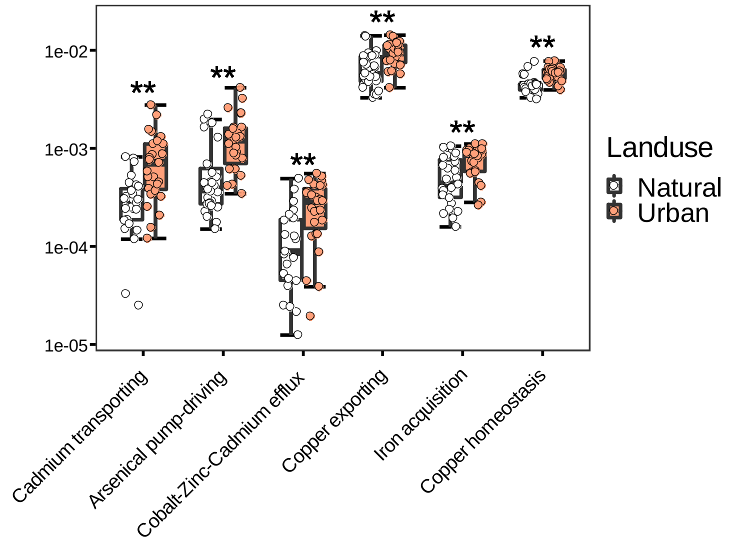

Figure S15. The genes are associated with the transport of metal(loid)s in the soils of urban greenspaces and adjacent natural areas. Asterisks indicate significant differences in nested PERMANOVA analyses using a block design as described in the Method section. ***P* < 0.01.

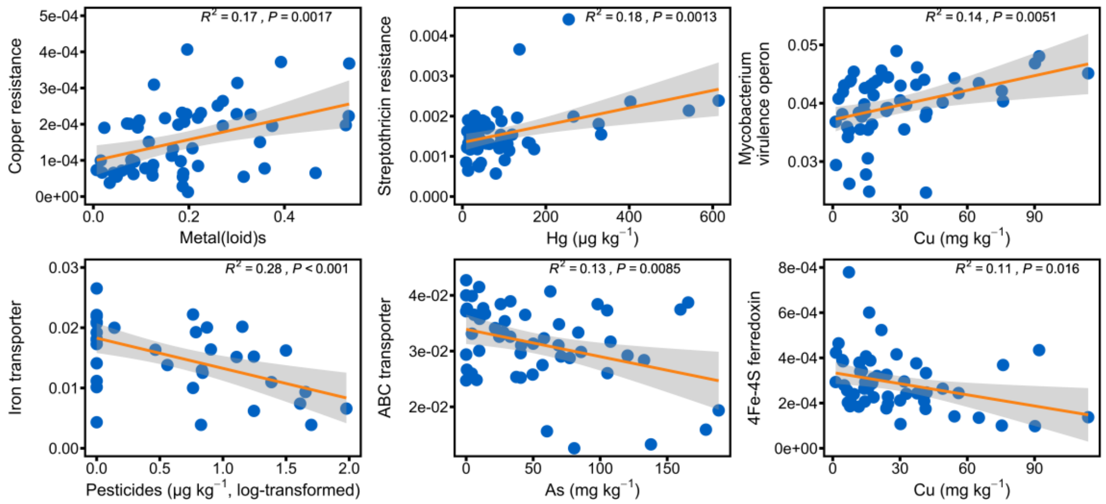

Figure S16. Relationships between soil contaminants and selected microbial functional genes. These functional genes are associated with environmental stress, pathogenicity, nutrient cycling and microbial metabolisms (see Suppmentary Table 9), which were annotated according to BacMet and MG-RAST using metagenomic data (see details in Methods).

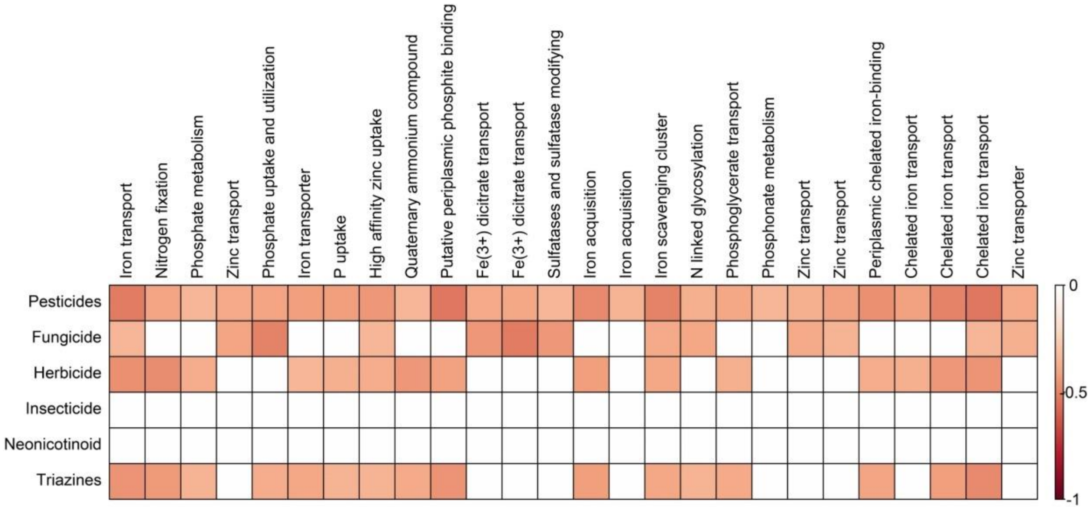

Figure S17. Spearman correlations between soil pesticide residues and the proportions of the selected genes associated with element cycles. Statistically non-significant correlations (*P* > 0.05) are shown in white.

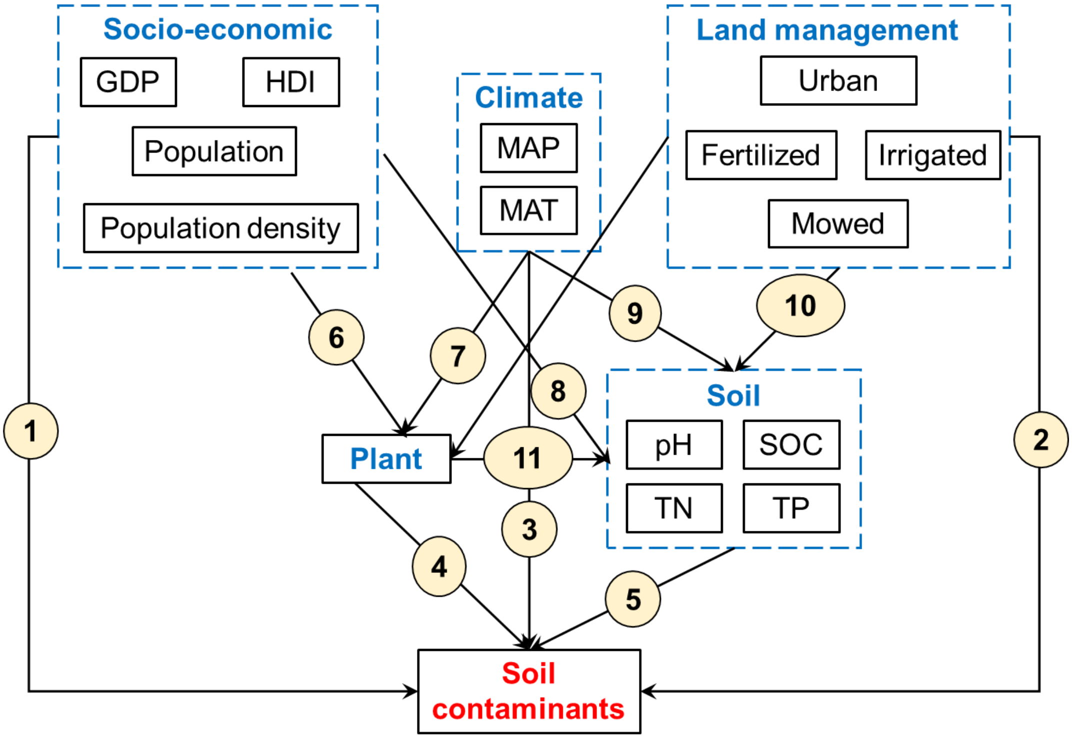

#	Associations	Rationale
1	Socio-economic<br>→<br>Soil contaminants	Socio-economic development is known to associated with releases of contaminants. For example, high population density and GPD are reported to be positively correlated with levels of contaminants in surrounding soils.
2	Land management<br>→<br>Soil contaminants	Land management influences the accumulation of soil contaminants. For example, fertilization and irrigation are known to be important sources of microplastics, ARGs, metal(loid)s and pesticides. Mowing can affect soil processes including transfer of contaminants.
3	Climate<br>→<br>Soil contaminants	Climate is known to influence the aerial transport of contaminants. For example, increases in MAP can lead to enhanced soil contamination via wet deposition.
4	Plant<br>→<br>Soil contaminants	Plant has interception effect on the contaminants from atmospheric deposition, and thus mitigate contaminant accumulation in soil.
5	Soil<br>→<br>Contaminants	Soil organic matter can bind to contaminants. Also, soil nutrients can influence soil microbes and thus microbial transformation of contaminants
6	Socio-economic<br>→<br>Plant	Socio-economic development is known to have disturbances on plants. For example, increased industry and human activities can adversely influence plant growth.

7	Climate → Plant	Climate regulates plant attributes. For example, MAP and MAT is expected to be positively associated with plant cover and richness.
8	Socio-economic → Soil	Lots of nutrients released from industries and domestic sewage could enhance their level in surrounding environments. For example, socio-economic development is known to positive associated with the levels of soil C, N and P.
9	Climate → Soil	Climate influences soil chemical properties. For example, increases in humidity (e.g. tropical rainforest) are known to be positively associated with soil nutrients. MAT is expected to have a positive effect on soil C.
10	Land management → Soil	Management practices such as fertilization, irrigation, and mowing could directly and indirectly influence soil fertility.
11	Plant → Soil	Plants are known to be closely associated with multiple soil properties. For example, increased plant cover is known to promote soil nutrients via litterfall.

Figure S18. A *priori* model identifying associations of socio-economic and environmental factors with contaminants in the soils of surveyed sites worldwide. The examined variables include socio-economic (population size and density, human development index (HDI), gross domestic product (GDP)) and (climate, plant and soil) accounting for different management practices (irrigated, mowed and fertilized). Urban indicates urban greenspaces, and plant represents plant cover. MAT, mean annual temperature; MAP, mean annual precipitation; SOC, soil organic carbon; TP, total phosphorus; TN, total nitrogen. Urban, urban greenspaces; Plant, plant cover.

Table S1. Detailed information on geographical coordinates of sampling sites and soil physichimcal properties in 56 locations across the globe.

Location	City	Landuse	Latitude (º)	Longitude (º)	TN (%)	TP (%)	SOC (g kg^{-1})	pH	Salinity (g kg^{-1})	Soil type	Vegetation	Climate
1	Tonghua City, Jilin,	Natural	41.7353	126.0447	0.57	0.05	13.24	5.72	171.36	Haplic Luvisols	Forest	Continental
	China	Urban	41.7394	125.9428	0.44	0.04	15.69	5.07	209.57			
2	Baishan City, Jilin,	Natural	42.2753	127.4425	0.89	0.06	19.08	4.67	369.82	Haplic Luvisols	Grassland	Continental
	China	Urban	42.1753	127.5003	0.2	0.1	3.49	6.36	122.78			
3	Yanji City, Jilin,	Natural	42.8406	129.4636	0.66	0.06	13.7	6.58	229.83	Haplic Luvisols	Forest	Continental
	China	Urban	42.9108	129.49	0.16	0.02	3.74	6.52	81.58			
4	Dunhua City, Jilin,	Natural	43.3389	128.2375	0.4	0.04	8.86	5.31	104.21	Haplic Phaeozems	Forest	Continental
	China	Urban	43.3808	128.2242	0.54	0.05	11.64	4.85	212.82			
5	Jilin City, Jilin,	Natural	43.8778	126.6067	0.69	0.07	14.81	6.58	217.27	Haplic Luvisols	Shrubland	Continental
	China	Urban	43.8425	126.5231	0.33	0.04	7.69	5.54	141.44			
6	Santiago, Metropolitan Region,	Natural	-33.3822	-70.9635	0.27	0.07	4.69	7.95	302.36	Luvic Calcisols	Forest	Temperate
	Chile	Urban	-33.3739	-70.6074	0.33	0.09	5.14	7.99	382.92			
7	Belo Horizonte, Minas Gerais State, Brazil	Natural	-19.2689	-43.585	0.09	0.01	1.39	5.09	46.24	Ferralic Cambisols	Grassland	Tropical
		Urban	-19.8737	-43.9726	0.15	0.03	4.43	7	119.76			
8	Contagem, Minas Gerais State,	Natural	-19.2653	-43.5836	0.09	0.01	1.45	5.1	50.39	Ferralic Cambisols	Forest	Tropical
	Brazil	Urban	-19.9399	-44.039	0.19	0.05	4.89	7.88	155.6			
9	Betim, Minas Gerais State,	Natural	-19.2944	-43.6058	0.2	0.01	3.52	4.38	59.05	Ferralic Cambisols	Grassland	Tropical
	Brazil	Urban	-19.9392	-44.1845	0.09	0.01	2	5.77	53.03			
10	Longmont, CO, USA	Natural	40.242	-105.212	0.15	0.02	2.7	6.13	135.66	Albic Luvisols	Grassland	Arid
		Urban	40.1614	-105.121	0.42	0.07	9.11	7.59	561.69			
11	Grand Junction, CO, USA	Natural	39.1904	-109.115	0.18	0.03	2.23	8.57	491.34	Eutric Fluvisols	Grassland	Arid
		Urban	39.1126	-108.608	0.26	0.07	3.89	8.62	222.45			

12	Cheyenne, WY, USA	Natural	41.3159	-104.827	0.15	0.02	2.76	7.46	70.37	Luvic Kastanozems	Grassland	Arid
		Urban	41.156	-104.831	0.33	0.04	5.69	7.95	188.83			
13	South Lyon, MI, USA	Natural	42.4451	-83.4423	0.32	0.03	5.11	7.98	139.94	Orthic Luvisols	Grassland	Continental
		Urban	42.4394	-83.6751	0.25	0.03	4.66	7.84	98.26			
14	Oxford, England, UK	Natural	53.0798	-2.1869	0.64	0.06	10.77	7.44	224.29	Dystric Gleysols	Grassland	Temperate
		Urban	53.0798	-2.1869	0.61	0.08	12.52	7.81	163.96			
15	Bodø, Norway	Natural	67.3018	14.3901	0.73	0.06	14.79	5.35	122.84	Gleyic Arenosols	Grassland	Continental
		Urban	67.2795	14.3976	0.73	0.12	13.8	7.08	289.15			
16	Uppsala, Sweden	Natural	59.8521	17.5977	0.76	0.06	25.33	5.23	172.16	Dystric Cambisols	Grassland	Continental
		Urban	59.8574	17.6403	0.71	0.11	18.83	6.47	633.84			
17	Poitiers, France	Natural	46.5069	0.3717	0.19	0.01	5.26	5.46	71.05	Eutric Cambisols	Forest	Temperate
		Urban	46.5756	0.3353	0.52	0.14	9.56	7.76	232.44			
18	Niort, France	Natural	46.3039	-0.6844	0.65	0.09	12.01	7.81	281.24	Gleyic Solonchaks	Forest	Temperate
		Urban	46.325	-0.4672	0.33	0.15	5.63	7.79	147.37			
19	Tours, France	Natural	47.3356	0.7514	0.48	0.02	11.35	4.46	82.4	Haplic Luvisols	Forest	Temperate
		Urban	47.3964	0.6772	0.19	0.05	3.45	7.76	103.26			
20	Ljubljana, Slovenia	Natural	46.0522	14.4783	0.44	0.05	11.24	4.15	100.42	Dystric Cambisols	Forest	Continental
		Urban	46.0519	14.4799	0.39	0.05	6.9	7.1	191.67			
21	Koper, Slovenia	Natural	45.5261	13.7172	0.54	0.05	14.1	7.62	245.27	Calcaric Cambisols	Forest	Temperate
		Urban	45.5444	13.7312	0.58	0.07	12.46	7.47	275.87			
22	Maribor, Slovenia	Natural	46.5809	15.6363	0.27	0.03	6.3	5.46	92.72	Eutric Cambisols	Forest	Continental
		Urban	46.5652	15.6488	0.4	0.1	6.18	7.37	148.33			
23	Pretoria, South Africa	Natural	-25.7879	28.203	0.32	0.05	6.51	6.01	127.16	Lithic Leptosols	Forest	Temperate
		Urban	-25.7618	28.2203	0.32	0.12	5.94	6.66	258.81			
24	Germiston, South	Natural	-26.4834	28.2116	0.55	0.06	11.51	5.15	265.67	Lithic Leptosols	Grassland	Temperate

	Africa										
		Urban	-26.1643	28.131	0.39	0.07	6.06	6.35	186.29			
25	Cape Town, South Africa	Natural	-33.3338	18.2729	0.08	0.02	1.87	8.03	900.06	Albic Arenosols	Forest	Temperate
		Urban	-33.904	18.402	0.55	0.09	11.46	7.93	303.11			
26	Durgapur, West Bengal, India	Natural	23.535	87.338	0.07	0.01	1.74	6.18	57	Orthic Luvisols	Forest	Tropical
		Urban	23.535	87.338	0.12	0.01	2.19	6.51	101.33			
27	Mirzapur, Uttar Pradesh, India	Natural	25.1461	82.569	0.08	0.01	1.47	6.01	53.67	Orthic Luvisols	Grassland	Temperate
		Urban	25.1461	82.569	0.24	0.16	5.07	8.14	95			
28	Agra, Uttar Pradesh, India	Natural	27.1752	78.0098	0.13	0.07	1.52	8.35	105	Eutric Cambisols	Grassland	Arid
		Urban	27.1752	78.0098	0.15	0.08	1.87	8.36	404			
29	Beijing, China	Natural	40.0726	116.182	0.19	0.07	3.22	8.22	236.1	Calcaric Fluvisols	Forest	Continental
		Urban	40.0103	116.3886	0.2	0.05	2.51	8.28	165.7			
30	Tai'an, Shandong, China	Natural	36.208	117.1102	0.52	0.03	7.79	5.19	231.21	Haplic Luvisols	Forest	Continental
		Urban	36.2218	117.0188	0.17	0.04	3.45	7.67	161.55			
31	Tianjin, China	Natural	39.0753	117.1478	0.2	0.05	3.46	8.32	172.95	Calcic Gleysols	Forest	Continental
		Urban	39.0846	117.6862	0.14	0.06	2.84	8.41	156.9			
32	Ürümqi, Xinjiang, China	Natural	43.8342	88.0452	1.18	0.08	35.96	6.66	699.43	Eutric Leptosols	Grassland	Arid
		Urban	43.8308	87.657	0.75	0.09	10.13	8.27	337.77			
33	Alice Springs, Northern Territory, Australia	Natural	-23.8255	133.3547	0.08	0.01	1.93	6.24	57.16	Lithosols	Forest	Arid
		Urban	-23.7083	133.8746	0.24	0.06	3.42	8.53	237.69			
34	Brisbane, Queensland, Australia	Natural	-27.4922	153.4294	0.19	0	2.09	4.95	35.67	Orthic Podzols	Grassland	Temperate
		Urban	-27.4984	153.016	0.58	0.09	11.49	6.59	172.91			
35	Mildura, Victoria,	Natural	-34.1802	142.1729	0.59	0.04	8.44	6.72	312.85	Chromic	Grassland	Arid

	Australia									Vertisols		
		Urban	-34.1854	142.1696	0.51	0.04	9.99	7.97	280.9			
36	Cecil Hills, New South Wales,	Natural	-33.8812	150.8546	0.45	0.05	11.49	6.44	152.17	Chromic Luvisols	Grassland	Temperate
	Australia	Urban	-33.8814	150.8533	0.46	0.09	8.75	6.97	165.9			
37	Heathcote, New South Wales,	Natural	-34.0721	151.0012	0.13	0	8.22	4.59	112.21	Orthic Acrisols	Grassland	Temperate
	Australia	Urban	-34.0822	151.0126	0.16	0.04	4	6.92	184.76			
38	Barcelona, Catalunya, Spain	Natural	41.4768	2.1475	0.56	0.05	11.07	6.65	364.94	Calcaric Cambisols	Forest	Temperate
		Urban	41.416	2.1507	0.3	0.05	6.43	7.77	323.1			
39	Pullman, Washington, USA	Natural	46.7255	-116.923	0.35	0.06	4.43	6.25	195.84	Luvic Phaeozems	Shrubland	Continental
		Urban	46.7401	-117.175	0.52	0.08	8.51	6.89	343.48			
40	Corvallis, Oregon, USA	Natural	44.4965	-123.338	0.3	0.05	13.25	5.44	214.7	Humic Acrisols	Grassland	Temperate
		Urban	44.5304	-123.26	0.59	0.06	63.23	5.16	432.51			
41	Coyoacán, Mexico City, Mexico	Natural	19.603	-99.478	1.29	0.11	35.58	5.92	198.85	Eutric Regosols	Shrubland	Temperate
		Urban	19.3106	-99.1831	1.4	0.08	27.76	5.89	211.77			
42	Tlalpan, Mexico City, Mexico	Natural	19.6013	-99.4759	1.07	0.08	29.78	7	129.57	Eutric Regosols	Forest	Temperate
		Urban	19.2943	-99.1939	0.5	0.05	11.27	6.78	178.35			
43	Miguel Hidalgo, Mexico City,	Natural	19.081	-99.1701	0.98	0.08	25.06	5.27	138.3	Eutric Regosols	Forest	Temperate
	Mexico	Urban	19.4188	-99.1863	0.59	0.12	12.05	7.52	190.38			
44	Madrid, La Comunidad de Madrid,	Natural	40.5722	-4.1533	0.32	0.06	5.95	6.4	310.39	Dystric Regosols	Forest	Arid
	Spain	Urban	40.4106	-3.6903	0.15	0.04	3.42	8.63	211.43			
45	Esa-Odo, Osun state, Nigeria	Natural	7.757	4.828	0.2	0.01	3.09	5.59	116.87	Ferric Luvisols	Forest	Tropical
		Urban	7.756	4.811	0.12	0.02	1.34	5.12	178.57			
46	Obafemi Awolowo University, Ile-Ife,	Natural	7.616	4.414	0.2	0.02	3.81	5.82	122.62	Ferric Luvisols	Forest	Tropical

	Osun state, Nigeria											
		Urban	7.517	4.528	0.2	0.02	3.7	6.82	139.05			
47	Ife city, Osun state, Nigeria	Natural	7.615	4.385	0.25	0.02	4.55	6.1	69.51	Ferric Luvisols	Shrubland	Tropical
		Urban	7.492	4.585	0.1	0.02	1.82	7.98	151.15			
48	Lakeland, Florida, USA	Natural	28.296	-81.0404	0.08	0.01	1.19	6.87	64.08	Gleyic Podzols	Forest	Temperate
		Urban	28.0404	-81.9666	0.19	0.02	1.86	8.26	237.8			
49	Sebring, Florida, USA	Natural	27.4712	-81.5303	0.11	0	1.9	7.17	76.52	Gleyic Podzols	Forest	Temperate
		Urban	27.4834	-81.4212	0.08	0.01	1.82	7.67	165.53			
50	Punta Gorda, Florida, USA	Natural	26.9093	-82.0678	0.05	0.02	0.78	7.41	101.21	Dystric Gleysols	Forest	Temperate
		Urban	26.9335	-82.0562	0.03	0.03	0.26	7.31	201.04			
51	Utrera, Sevilla, Spain	Natural	37.2011	-5.8445	0.29	0.01	1.74	5.71	88.73	Calcaric Cambisols	Forest	Temperate
		Urban	37.1929	-5.7666	0.13	0.07	2.14	8.21	119.8			
52	Coimbra, Portugal	Natural	40.0956	-8.4935	0.26	0.03	10.4	6.11	77.58	Calcaric Cambisols	Forest	Temperate
		Urban	40.2052	-8.4206	0.06	0.02	1.24	8.31	58.59			
53	Porto, Portugal	Natural	41.1572	-8.485	1.56	0.06	47.97	3.98	352.1	Humic Cambisols	Forest	Temperate
		Urban	41.1672	-8.679	0.33	0.05	6.7	5.39	216.43			
54	Jerusalem, Israel	Natural	31.752	35.1292	0.15	0.02	3.12	7.93	140.21	Chromic Luvisols	Forest	Temperate
		Urban	31.7701	35.2235	0.6	0.18	12.44	8.02	423.47			
55	Be'er Sheva, Israel	Natural	31.2493	34.7415	0.15	0.03	1.51	8.44	135.03	Calcic Yermosols	Forest	Arid
		Urban	31.2347	34.7927	0.12	0.03	2	8.63	192.4			
56	Ofakim, Israel	Natural	31.2711	34.6503	0.08	0.02	1.06	8.6	92.61	Calcaric Regosols	Forest	Arid
		Urban	31.3095	34.6307	0.13	0.04	2.63	8.38	358.32			

Table S2. The soil contaminants including metal(loid)s, pesticides, microplastics (MPs), and antibiotic resistance genes (ARGs) determined in different sites worldwide. Yes and No indicates that the soil contaminants were determined or not in the location, respectively.

Location	Metal(loid)s	ARGs	Pesticides	MPs
1	Yes	Yes	No	No
2	Yes	Yes	No	No
3	Yes	Yes	No	No
4	Yes	Yes	Yes	Yes
5	Yes	Yes	No	No
6	Yes	Yes	Yes	Yes
7	Yes	Yes	Yes	Yes
8	Yes	Yes	No	No
9	Yes	Yes	No	No
10	Yes	Yes	No	No
11	Yes	Yes	No	No
12	Yes	Yes	No	No
13	Yes	Yes	No	No
14	Yes	Yes	Yes	Yes
15	Yes	Yes	Yes	Yes
16	Yes	Yes	No	Yes
17	Yes	Yes	No	No
18	Yes	Yes	No	No
19	Yes	Yes	Yes	Yes
20	Yes	Yes	Yes	Yes
21	Yes	Yes	No	No
22	Yes	Yes	Yes	Yes
23	Yes	Yes	Yes	Yes
24	Yes	Yes	Yes	Yes
25	Yes	Yes	Yes	Yes
26	Yes	Yes	Yes	Yes
27	Yes	Yes	No	No
28	Yes	Yes	No	No
29	Yes	Yes	Yes	Yes
30	Yes	Yes	No	No
31	Yes	Yes	No	No
32	Yes	Yes	No	Yes
33	Yes	Yes	Yes	Yes
34	Yes	Yes	Yes	Yes
35	Yes	Yes	No	No
36	Yes	Yes	No	No
37	Yes	Yes	Yes	Yes
38	Yes	Yes	Yes	Yes
39	Yes	Yes	Yes	Yes
40	Yes	Yes	No	No

41	Yes	Yes	No	Yes
42	Yes	Yes	No	Yes
43	Yes	Yes	Yes	No
44	Yes	Yes	Yes	Yes
45	Yes	Yes	Yes	Yes
46	Yes	Yes	No	Yes
47	Yes	Yes	No	Yes
48	Yes	Yes	Yes	No
49	Yes	Yes	No	Yes
50	Yes	Yes	Yes	Yes
51	Yes	Yes	Yes	Yes
52	Yes	Yes	No	No
53	Yes	Yes	Yes	Yes
54	Yes	Yes	Yes	Yes
55	Yes	Yes	No	Yes
56	Yes	Yes	Yes	No

Table S3. A list of antibiotic resistance genes (ARGs) mobile genetic elements (MGEs) considered in this study.

Gene Name	Type	Gene Classification	Resistance Mechanism
16S rRNA	16S rRNA	16S rRNA	Internal control
aac	ARG	Aminoglycoside	deactivate
aacC1	ARG	Aminoglycoside	deactivate
aacC2	ARG	Aminoglycoside	deactivate
aacC4	ARG	Aminoglycoside	deactivate
aac(6')I1	ARG	Aminoglycoside	deactivate
aacA/aphD	ARG	Aminoglycoside	deactivate
aac(6')-Iy	ARG	Aminoglycoside	deactivate
aac(6')-II	ARG	Aminoglycoside	deactivate
aacC	ARG	Aminoglycoside	deactivate
aac(6')-Ib-01	ARG	Aminoglycoside	deactivate
aac(6')-Ib-02	ARG	Aminoglycoside	deactivate
aadA5-01	ARG	Aminoglycoside	deactivate
aadA5-02	ARG	Aminoglycoside	deactivate
aac(6')-Ib-03	ARG	Aminoglycoside	deactivate
aadA1	ARG	Aminoglycoside	deactivate
aadA2-01	ARG	Aminoglycoside	deactivate
aadA-01	ARG	Aminoglycoside	deactivate
aadA2-02	ARG	Aminoglycoside	deactivate
aadD	ARG	Aminoglycoside	deactivate
aadA2-03	ARG	Aminoglycoside	deactivate
aadA9-01	ARG	Aminoglycoside	deactivate
aadA-02	ARG	Aminoglycoside	deactivate
aadA9-02	ARG	Aminoglycoside	deactivate
aadE	ARG	Aminoglycoside	deactivate
spcN-01	ARG	Aminoglycoside	deactivate
spcN-02	ARG	Aminoglycoside	deactivate
aadA-1-01	ARG	Aminoglycoside	deactivate
aph6ia	ARG	Aminoglycoside	deactivate
aph(2')-Id-02	ARG	Aminoglycoside	deactivate
aph(2')-Id-01	ARG	Aminoglycoside	deactivate
aph	ARG	Aminoglycoside	deactivate
aphA1	ARG	Aminoglycoside	deactivate
aadA-1-02	ARG	Aminoglycoside	deactivate
str	ARG	Aminoglycoside	deactivate
strA	ARG	Aminoglycoside	deactivate
strB	ARG	Aminoglycoside	deactivate
ampC-01	ARG	Beta Lactamase	deactivate
ampC-02	ARG	Beta Lactamase	deactivate
ampC-04	ARG	Beta Lactamase	deactivate
ampC-05	ARG	Beta Lactamase	deactivate
ampC-06	ARG	Beta Lactamase	deactivate
ampC-07	ARG	Beta Lactamase	deactivate
ampC-09	ARG	Beta Lactamase	deactivate
ampC/blaDHA	ARG	Beta Lactamase	deactivate

bla1	ARG	Beta Lactamase	deactivate
bla-ACC-1	ARG	Beta Lactamase	deactivate
blaCMY	ARG	Beta Lactamase	deactivate
blaCMY2-01	ARG	Beta Lactamase	deactivate
blaCMY2-02	ARG	Beta Lactamase	deactivate
blaCTX-M-01	ARG	Beta Lactamase	deactivate
blaCTX-M-02	ARG	Beta Lactamase	deactivate
blaCTX-M-03	ARG	Beta Lactamase	deactivate
blaCTX-M-04	ARG	Beta Lactamase	deactivate
blaCTX-M-05	ARG	Beta Lactamase	deactivate
blaCTX-M-06	ARG	Beta Lactamase	deactivate
blaGES	ARG	Beta Lactamase	deactivate
blaIMP-01	ARG	Beta Lactamase	deactivate
blaIMP-02	ARG	Beta Lactamase	deactivate
bla-L1	ARG	Beta Lactamase	deactivate
blaMOX/blaCMY	ARG	Beta Lactamase	deactivate
blaOCH	ARG	Beta Lactamase	deactivate
blaOKP	ARG	Beta Lactamase	deactivate
blaOXA1/blaOXA30	ARG	Beta Lactamase	deactivate
blaOXA10-01	ARG	Beta Lactamase	deactivate
blaOXA10-02	ARG	Beta Lactamase	deactivate
blaOXY	ARG	Beta Lactamase	deactivate
blaPAO	ARG	Beta Lactamase	deactivate
blaPER	ARG	Beta Lactamase	deactivate
blaPSE	ARG	Beta Lactamase	deactivate
blaROB	ARG	Beta Lactamase	deactivate
blaSFO	ARG	Beta Lactamase	deactivate
blaSHV-01	ARG	Beta Lactamase	deactivate
blaSHV-02	ARG	Beta Lactamase	deactivate
blaTEM	ARG	Beta Lactamase	deactivate
blaTLA	ARG	Beta Lactamase	deactivate
blaVEB	ARG	Beta Lactamase	deactivate
blaVIM	ARG	Beta Lactamase	deactivate
blaZ	ARG	Beta Lactamase	deactivate
cepA	ARG	Beta Lactamase	deactivate
cfiA	ARG	Beta Lactamase	deactivate
cfxA	ARG	Beta Lactamase	deactivate
cphA-01	ARG	Beta Lactamase	deactivate
cphA-02	ARG	Beta Lactamase	deactivate
fox5	ARG	Beta Lactamase	deactivate
mecA	ARG	Beta Lactamase	protection
NDM1	ARG	Beta Lactamase	deactivate
pbp	ARG	Beta Lactamase	protection
pbp2x	ARG	Beta Lactamase	protection
Pbp5	ARG	Beta Lactamase	protection
penA	ARG	Beta Lactamase	protection
carB	ARG	MLSB	efflux
ereA	ARG	MLSB	deactivate
ereB	ARG	MLSB	deactivate
erm(34)	ARG	MLSB	protection
erm(35)	ARG	MLSB	protection

erm(36)	ARG	MLSB	protection
ermA	ARG	MLSB	protection
ermA/ermTR	ARG	MLSB	protection
ermB	ARG	MLSB	protection
ermC	ARG	MLSB	protection
ermF	ARG	MLSB	protection
ermJ/ermD	ARG	MLSB	protection
ermK-01	ARG	MLSB	protection
ermK-02	ARG	MLSB	protection
ermT-01	ARG	MLSB	protection
ermT-02	ARG	MLSB	protection
ermX	ARG	MLSB	protection
ermY	ARG	MLSB	protection
lmrA-01	ARG	MLSB	efflux
lnuA-01	ARG	MLSB	deactivate
lnuB-01	ARG	MLSB	deactivate
lnuB-02	ARG	MLSB	deactivate
lnuC	ARG	MLSB	deactivate
matA/mel	ARG	MLSB	efflux
mdtA	ARG	MLSB	efflux
mefA	ARG	MLSB	efflux
mphA-01	ARG	MLSB	deactivate
mphA-02	ARG	MLSB	deactivate
mphB	ARG	MLSB	deactivate
mphC	ARG	MLSB	deactivate
msrA-01	ARG	MLSB	efflux
msrC-01	ARG	MLSB	efflux
oleC	ARG	MLSB	efflux
pikR1	ARG	MLSB	protection
pikR2	ARG	MLSB	protection
vatB-01	ARG	MLSB	deactivate
vatB-02	ARG	MLSB	deactivate
vatC-01	ARG	MLSB	deactivate
vatC-02	ARG	MLSB	deactivate
vatE-01	ARG	MLSB	deactivate
vatE-02	ARG	MLSB	deactivate
vgaA-01	ARG	MLSB	efflux
vgaA-02	ARG	MLSB	efflux
vgb-01	ARG	MLSB	deactivate
vgbB-01	ARG	MLSB	efflux
vgbB-02	ARG	MLSB	deactivate
sul2	ARG	Sulfonamide	protection
sul1	ARG	Sulfonamide	protection
sulA/folP-01	ARG	Sulfonamide	protection
sulA/folP-03	ARG	Sulfonamide	protection
dfrA1	ARG	Sulfonamide	deactivate
dfrA12	ARG	Sulfonamide	deactivate
folA	ARG	Sulfonamide	deactivate
tet(32)	ARG	Tetracycline	protection
tet(34)	ARG	Tetracycline	unknown
tet(35)	ARG	Tetracycline	unknown

tet(36)-01	ARG	Tetracycline	protection
tet(36)-02	ARG	Tetracycline	protection
tet(37)	ARG	Tetracycline	unknown
tetA-01	ARG	Tetracycline	efflux
tetA-02	ARG	Tetracycline	efflux
tetB-01	ARG	Tetracycline	efflux
tetB-02	ARG	Tetracycline	efflux
tetC-01	ARG	Tetracycline	efflux
tetC-02	ARG	Tetracycline	efflux
tetD-01	ARG	Tetracycline	efflux
tetD-02	ARG	Tetracycline	efflux
tetE	ARG	Tetracycline	efflux
tetG-01	ARG	Tetracycline	efflux
tetG-02	ARG	Tetracycline	efflux
tetH	ARG	Tetracycline	efflux
tetJ	ARG	Tetracycline	efflux
tetK	ARG	Tetracycline	efflux
tetL-01	ARG	Tetracycline	efflux
tetL-02	ARG	Tetracycline	efflux
tetM-01	ARG	Tetracycline	protection
tetM-02	ARG	Tetracycline	protection
tetO-01	ARG	Tetracycline	protection
tetPA	ARG	Tetracycline	efflux
tetPB-01	ARG	Tetracycline	protection
tetPB-02	ARG	Tetracycline	protection
tetPB-03	ARG	Tetracycline	protection
tetPB-05	ARG	Tetracycline	protection
tetQ	ARG	Tetracycline	protection
tetR-02	ARG	Tetracycline	efflux
tetR-03	ARG	Tetracycline	efflux
tetS	ARG	Tetracycline	protection
tetT	ARG	Tetracycline	protection
tetU-01	ARG	Tetracycline	unknown
tetV	ARG	Tetracycline	efflux
tetW-01	ARG	Tetracycline	protection
tetX	ARG	Tetracycline	unknown
vanA	ARG	Vancomycin	protection
vanB-01	ARG	Vancomycin	protection
vanC-01	ARG	Vancomycin	protection
vanC-02	ARG	Vancomycin	protection
vanC-03	ARG	Vancomycin	protection
vanC1	ARG	Vancomycin	protection
vanC2/vanC3	ARG	Vancomycin	protection
vanG	ARG	Vancomycin	protection
vanHB	ARG	Vancomycin	protection
vanHD	ARG	Vancomycin	protection
vanRA-01	ARG	Vancomycin	protection
vanRA-02	ARG	Vancomycin	protection
vanRB	ARG	Vancomycin	protection
vanRC	ARG	Vancomycin	protection
vanRC4	ARG	Vancomycin	protection

vanRD	ARG	Vancomycin	protection
vanSA	ARG	Vancomycin	protection
vanSB	ARG	Vancomycin	protection
vanSC-01	ARG	Vancomycin	protection
vanSC-02	ARG	Vancomycin	protection
vanTC-01	ARG	Vancomycin	protection
vanTC-02	ARG	Vancomycin	protection
vanTE	ARG	Vancomycin	protection
vanTG	ARG	Vancomycin	protection
vanWB	ARG	Vancomycin	protection
vanWG	ARG	Vancomycin	protection
vanXA	ARG	Vancomycin	protection
vanXB	ARG	Vancomycin	protection
vanXD	ARG	Vancomycin	protection
vanYB	ARG	Vancomycin	protection
vanYD-01	ARG	Vancomycin	protection
vanYD-02	ARG	Vancomycin	protection
acrA-01	ARG	Multidrug	efflux
acrA-02	ARG	Multidrug	efflux
acrA-03	ARG	Multidrug	efflux
acrA-04	ARG	Multidrug	efflux
acrB-01	ARG	Multidrug	efflux
acrR-01	ARG	Multidrug	efflux
acrR-02	ARG	Multidrug	efflux
adeA	ARG	Multidrug	efflux
acrA-05	ARG	Multidrug	efflux
acrF	ARG	Multidrug	efflux
ceoA	ARG	Multidrug	efflux
cmeA	ARG	Multidrug	efflux
cmr	ARG	Multidrug	efflux
emrD	ARG	Multidrug	efflux
marR-01	ARG	Multidrug	efflux
mdetl1	ARG	Multidrug	efflux
mdtE/yhiU	ARG	Multidrug	efflux
mepA	ARG	Multidrug	efflux
mexA	ARG	Multidrug	efflux
mexD	ARG	Multidrug	efflux
mexE	ARG	Multidrug	efflux
mexF	ARG	Multidrug	efflux
mtrC-01	ARG	Multidrug	efflux
mtrC-02	ARG	Multidrug	efflux
mtrD-02	ARG	Multidrug	efflux
mtrD-03	ARG	Multidrug	efflux
oprD	ARG	Multidrug	efflux
oprJ	ARG	Multidrug	efflux
pmrA	ARG	Multidrug	efflux
putative multidrug	ARG	Multidrug	efflux
qac	ARG	Multidrug	efflux
qacA	ARG	Multidrug	efflux
qacA/qacB	ARG	Multidrug	efflux
qacEdelta1-01	ARG	Multidrug	efflux

qacEdelta1-02	ARG	Multidrug	efflux
qacH-01	ARG	Multidrug	efflux
qacH-02	ARG	Multidrug	efflux
rarD-02	ARG	Multidrug	efflux
sdeB	ARG	Multidrug	efflux
tolC-01	ARG	Multidrug	efflux
tolC-02	ARG	Multidrug	efflux
tolC-03	ARG	Multidrug	efflux
ttgA	ARG	Multidrug	efflux
ttgB	ARG	Multidrug	efflux
yceE/mdtG-01	ARG	Multidrug	efflux
yceE/mdtG-02	ARG	Multidrug	efflux
yceL/mdtH-01	ARG	Multidrug	efflux
yceL/mdtH-02	ARG	Multidrug	efflux
yceL/mdtH-03	ARG	Multidrug	efflux
yidY/mdtL-01	ARG	Multidrug	efflux
yidY/mdtL-02	ARG	Multidrug	efflux
fabK	ARG	other	deactivate
imiR	ARG	other	unknown
nisB	ARG	other	unknown
speA	ARG	other	unknown
bacA-01	ARG	other	deactivate
bacA-02	ARG	other	deactivate
fosB	ARG	other	deactivate
fosX	ARG	other	deactivate
nimE	ARG	other	unknown
pncA	ARG	other	unknown
sat4	ARG	other	deactivate

Table S4. A statistical summary of nest-PERMANOVA of soil contaminants between paired urban greenspaces and adjacent natural areas across the globe.

		Df	Mean	F	R^2	Pr(>F)
Multi-contamination	Landuse	1	0.007	1.31	0.03	0.15
	Residuals	46	0.005	0.97		
	Total	47	1.00			
Metal(loid)s	Landuse	1	0.05	3.56	0.03	0.01
	Residuals	110	0.01	0.97		
	Total	111	1.00			
As	Landuse	1	620	0.24	0.00	0.48
	Residuals	110	2541	1.00		
	Total	111	1.00			
Cd	Landuse	1.00	0.13	1.25	0.01	0.10
	Residuals	110	0.10	0.99		
	Total	111	1.00			
Pb	Landuse	1	2142	1.78	0.02	0.10
	Residuals	110	1205	0.98		
	Total	111	1.00			
Cr	Landuse	1	3026	1.13	0.01	0.06
	Residuals	110	2668	0.99		
	Total	111	1.00			
Cu	Landuse	1	3597	5.90	0.05	0.00
	Residuals	110	609	0.95		
	Total	111	1.00			
Ni	Landuse	1	89.2	0.26	0.00	0.36
	Residuals	110	346	1.00		
	Total	111	1.00			
Zn	Landuse	1	138962	6.64	0.06	0.00
	Residuals	110	20934	0.94		
	Total	111	1.00			
Hg	Landuse	1	46200	4.81	0.04	0.01
	Residuals	110	9603	0.96		
	Total	111	1.00			
Pesticides	Landuse	1	169	0.04	0.00	0.64
	Residuals	52	4136	1.00		
	Total	53	1.00			
Atrazine	Landuse	1	70.88	0.83	0.02	0.17
	Residuals	52	85.04	0.98		
	Total	53	1.00			
Fungicide	Landuse	1	0.50	0.00	0.00	0.99
	Residuals	52	196	1.00		
	Total	53	1.00			
Herbicide	Landuse	1	96.5	0.52	0.01	0.19

	Residuals	52	186	0.99		
	Total	53	1.00			
Insecticide	Landuse	1	490	0.18	0.00	0.13
	Residuals	52	2801	1.00		
	Total	53	1.00			
MPs	Landuse	1	1.30	2.39	0.04	0.06
	Residuals	62	0.54	0.96		
	Total	63	1.00			
Fragments	Landuse	1	16.3	0.02	0.00	0.87
	Residuals	62	820	1.00		
	Total	63	1.00			
Films	Landuse	1	149	0.41	0.01	0.57
	Residuals	62	362	0.99		
	Total	63	1.00			
Fibers	Landuse	1	67.1	0.07	0.00	0.81
	Residuals	62	967	1.00		
	Total	63	1.00			
PP	Landuse	1	162	0.21	0.00	0.66
	Residuals	62	763.	1.00		
	Total	63	1.00			
Polyester	Landuse	1	64.5	0.09	0.00	0.74
	Residuals	62	704	1.00		
	Total	63	1.00			
HDPE	Landuse	1	0.52	0.01	0.00	1.00
	Residuals	62	54.4	1.00		
	Total	63	1.00			
LDPE	Landuse	1	15.8	0.05	0.00	0.84
	Residuals	62	333	1.00		
	Total	63	1.00			
ARG	Landuse	1	0.02	0.22	0.00	0.62
	Residuals	110	0.08	1.00		
	Total	111	1.00			
Multidrug	Landuse	1	0.00	0.06	0.00	0.79
	Residuals	110	0.04	1.00		
	Total	111	1.00			
MLSB_abundance	Landuse	1	0.00	1.03	0.01	0.40
	Residuals	110	0.00	0.99		
	Total	111	1.00			
Tetracycline	Landuse	1	0.00	0.29	0.00	0.59
	Residuals	110	0.00	1.00		
	Total	111	1.00			
Vancomycin	Landuse	1	0.00	0.16	0.00	0.68
	Residuals	110	0.00	1.00		
	Total	111	1.00			

Aminoglycoside	Landuse	1	0.00	0.02	0.00	0.84
	Residuals	110	0.00	1.00		
	Total	111	1.00			
Beta_Lactamase	Landuse	1	0.00	0.36	0.00	0.47
	Residuals	110	0.00	1.00		
	Total	111	1.00			
Sulfonamide	Landuse	1	0.00	2.37	0.02	0.02
	Residuals	110	0.00	0.98		
	Total	111	1.00			
Others	Landuse	1	0.00	0.00	0.00	0.97
	Residuals	110	0.00	1.00		
	Total	111	1.00			
Richness of ARGs	Landuse	1	390	1.12	0.01	0.04
	Residuals	110	348.	0.99		
	Total	111	1.00			

Table S5. Average levels of soil contaminants in urban greenspaces and adjacent natural areas. For the contents of and pesticide residues, the unit is μg kg^{-1}, while for metal(loid)s, the unit is mg kg^{-1}. Content of microplastics (MPs) was shown as item g^{-1}, and the shape and material composition are present as %. ARGs are showed as relative abundances (see Methods). *Pesticide transformation products. [a] means ± standard error.

**Contaminants**	**Implement**	**Natural areas**	**Urban greenspaces**
As	Metal(loid)	48.38 ± 3.91[a]	53.09. ± 3.86
Cd	Metal(loid)	0.41 ± 0.022	0.48 ± 0.03
Cr	Metal(loid)	43.75 ± 3.10	54.14 ± 4.71
Cu	Metal(loid)	18.18 ± 1.36	29.51. ± 2.32
Ni	Metal(loid)	18.95± 1.51	20.75. ± 1.36
Pb	Metal(loid)	37.53 ± 2.48	46.27 ± 2.87
Zn	Metal(loid)	55.44 ± 3.35	125.89 ± 15.43
Hg	Metal(loid)	0.063 ± 0.004	0.103 ± 0.097
6-Deisopropylatrazine*	Herbicide	0.011 ± 0.01	0.24 ± 0.08
Clothianidin	Insecticide	3.01 ± 1.68	1.63 ± 0.94
2,6-Dichlorbenzamid*	Herbicide	0	0.29 ± 0.12
Deethylatrazine	Herbicide	0	0
Thiamethoxam	Insecticide	0.73 ± 0.39	0.18 ± 0.10
2-Hydroxyatrazine*	Herbicide	5.53 ± 1.08	2.95 ± 0.94
Carbendazim	Fungicide	0.52 ± 0.21	0.18 ± 0.10
Chloridazon	Herbicide	0	0
Metamitron	Herbicide	0	0
Imidacloprid	Insecticide	3.92 ± 2.13	11.42 ± 6.14
Terbutylazine-desethyl*	Herbicide	0.02 ± 0.01	0
1-(4-Isopropylphenyl)urea*	Herbicide	0	0
Diuron	Herbicide	2.10 ± 1.21	2.36 ± 1.20
Atrazine	Herbicide	0.05 ± 0.01	0.12 ± 0.04
Thiacloprid	Insecticide	0	0.43 ± 0.25
Isoproturon	Herbicide	0	0
Linuron	Herbicide	0	0
Fludioxonil	Fungicide	0	0
Terbutylazine	Herbicide	0.05 ± 0.03	0
Pirimicarb	Insecticide	0	0
Fluxapyroxad	Fungicide	0.40 ± 0.22	0
Fluopyram	Fungicide	0.02 ± 0.01	0.02 ± 0.01
Cyproconazole	Fungicide	0.02 ± 0.01	0
Metalaxyl	Fungicide	0.16 ± 0.09	0.14 ± 0.08
Ethofumesate	Herbicide	0	0
Fluopicolid	Fungicide	0	0
Boscalid	Fungicide	0	0
Tebuconazole	Fungicide	0.27 ± 0.12	2.02 ± 1.11
Flusilazole	Fungicide	0	0

Acetochlor	Herbicide	0	0
Pyrifenox	Fungicide	0	0
Cyprodinil	Fungicide	0	0
Epoxiconazole	Fungicide	0	0
Alachlor	Herbicide	0	0
S-Metolachlor	Herbicide	0	0
Napropamide	Herbicide	0	0
Propiconazole	Fungicide	0.65 ± 0.21	1.57 ± 0.90
Azoxystrobin	Fungicide	1.93 ± 0.76	0.23 ± 0.13
Orbencarb	Herbicide	0	0
Trifloxystrobin	Fungicide	0	0
Fenpropidin	Fungicide	0	0
Chlorpyrifos	Insecticide	0.09 ± 0.05	0.13 ± 0.06
Metolachlor-OA*	Herbicide	0.86 ± 0.50	0
Metolachlor-ESA*	Herbicide	0	0
Fipronil	Insecticide	0	0
Oryzalin	Herbicide	0	0
PP (%)	MPs	59.16 ± 2.94	55.97 ± 2.70
Polyester (%)	MPs	31.63 ± 2.69	33.64 ± 2.73
HDPE (%)	MPs	1.56 ± 0.90	1.74 ± 0.57
LDPE (%)	MPs	7.65 ± 1.68	8.64± 2.04
Fibers (%)	MPs	69.41 ± 3.30	71.46 ± 3.05
Fragments (%)	MPs	18.04 ± 2.96	19.05 ± 2.88
Film (%)	MPs	12.55 ± 2.30	9.50 ± 1.51
Aminoglycoside	ARGs	0.012 ± 0.001	0.011 ± 0.001
Beta Lactamase	ARGs	0.051 ± 0.004	0.046 ± 0.003
FCA	ARGs	0.002 ± 0.0005	0
MLSB	ARGs	0.014 ± 0.003	0.008 ± 0.001
Multidrug	ARGs	0.171 ± 0.017	0.162 ± 0.013
Other	ARGs	0.002 ± 0.0002	0.002 ± 0.0002
Sulfonamide	ARGs	0	0
Tetracycline	ARGs	0.012 ± 0.001	0.011 ± 0.001
Vancomycin	ARGs	0.011 ± 0.001	0.01 ± 0.001
ARGs Richness	ARGs	68.786 ± 1.443	72.518 ± 1.438

Table S6. Percentages of soil samples exceeding lower guideline values of Metal(loid) thresholds of Finnish Legislation [3].

Metal(loid)s	Lower guideline value (mg kg^{-1})	% exceeding the limit (Urban)	% exceeding the limit (Natural)
Arsenic (As)	50	42.2	35.7
Mercury (Hg)	2	0	0
Cadmium (Cd)	10	0	0
Chrome (Cr)	200	3.6	1.8
Copper (Cu)	150	1.8	0
Lead (Pb)	200	1.8	0
Nickel (Ni)	100	0	0
Zinc (Zn)	250	8.9	0

Table S7. Spearman correlations between socio-economic and environmental factors and the response ratios of soil contaminants in urban greenspaces compared with adjacent natural areas. MPs, microplastics; ARGs, antibiotic resistance genes. HDI, human development index; GDP, gross domestic product. Urban indicates urban greenspaces, and plant represents plant cover. MAT, mean annual temperature; MAP, mean annual precipitation.*P < 0.05; **P < 0.01.

	**Metal(loid)s**	**Pesticides**	**MPs**	**ARGs**
HDI	0.05	-0.06	-0.13	-0.22
GDP	0.08	-0.01	-0.07	-0.22
Population	0.09	-0.22	0.09	0.04
Population density	0.11	0.16	**0.41***	-0.11
Irrigated	0.14	0.34	-0.17	**-0.36****
Fertilized	0.02	0.29	0.12	-0.07
Mowed	0.18	0.21	0.03	**-0.49****
MAT	0.1	0.38	-0.14	-0.23
MAP	0.07	-0.08	-0.04	-0.15
Plant cover	0.15	0.03	-0.11	-0.31*
Total N	0.26	-0.3	**0.38***	-0.22
Total P	**0.36****	-0.09	0.16	**-0.31***
Organic carbon	0.26	-0.25	**0.36***	-0.18
pH	-0.08	0.17	-0.19	-0.16

Table S8. A statistical summary of nest-PERMANOVA of genes associated with metal(loid) resistance between paired urban greenspaces and adjacent natural areas across the globe.

		Df	Mean	F	R^2	Pr(>F)
As	Landuse	1	1.41E-08	7.69	0.13	0.01
	Residuals	52	1.83E-09	0.87		
	Total	53	1			
Cu	Landuse	1	2.82E-07	14.97	0.22	0.00
	Residuals	52	1.88E-08	0.78		
	Total	53	1			
Hg	Landuse	1	6.26E-09	6.56	0.11	0.00
	Residuals	52	9.55E-10	0.89		
	Total	53	1			
Cd	Landuse	1	1.66E-09	13.25	0.20	0.00
	Residuals	52	1.25E-10	0.80		
	Total	53	1			
Cr	Landuse	1	1.01E-07	6.70	0.11	0.01
	Residuals	52	1.51E-08	0.89		
	Total	53	1			
Pb	Landuse	1	1.38E-05	26.62	0.34	0.00
	Residuals	52	5.19E-07	0.66		
	Total	53	1			
Zn	Landuse	1	4.21E-07	2.94	0.05	0.04
	Residuals	52	1.43E-07	0.95		
	Total	53	1			
Ni	Landuse	1	1.09E-10	0.49	0.01	0.40
	Residuals	52	2.23E-10	0.99		
	Total	53	1			

Table S9. The resistance included the resistant genes to stresses of metal(loid), drugs and pathogens, and the pathogenesis was assessed by the abundance of selected pathogenic genes in the soil microbiome

Gene	Description	Function	Database
MGRUST44	Alanine biosynthesis	Stress resistance	MGRUST
MGRUST65	Anaerobic Oxidative Degradation of L Ornithine	Stress resistance	MGRUST
MGRUST115	Bacillus biofilm matrix protein component TasA and homologs	Stress resistance	MGRUST
MGRUST294	CMP N acetylneuraminate Biosynthesis	Stress resistance	MGRUST
MGRUST331	Copper homeostasis	Stress resistance	MGRUST
MGRUST399	DNA repair	Stress resistance	MGRUST
MGRUST470	Folate Biosynthesis	Stress resistance	MGRUST
MGRUST650	Mercury resistance	Stress resistance	MGRUST
MGRUST666	Methylglyoxal Metabolism	Stress resistance	MGRUST
MGRUST686	Multidrug Resistance Operon mdtRP of Bacillus	Stress resistance	MGRUST
MGRUST942	Oxidative stress	Stress resistance	MGRUST
MGRUST1033	SigmaB stress responce regulation	Stress resistance	MGRUST
MGRUST1128	Thiamin biosynthesis LDP	Stress resistance	MGRUST
MGRUST1202	Tryptophan synthesis	Stress resistance	MGRUST
MGRUST1208	Type 1 pili mannose sensitive fimbriae	Stress resistance	MGRUST
MGRUST1212	Type III secretion system orphans	Stress resistance	MGRUST
MGRUST464	Flagellum	Stress resistance	MGRUST
MGRUST463	Flagellar motility	Stress resistance	MGRUST
MGRUST890	Pseudouridine Metabolism	Stress resistance	MGRUST
MGRUST1073	Streptothricin resistance	Stress resistance	MGRUST
actP	Copper-transporting P-type ATPase	Stress resistance	BacMet
aioA/aoxB	Arsenite oxidase subunit	Stress resistance	BacMet
aioB/aoxA	Arsenite oxidase subunit	Stress resistance	BacMet
arsM	Arsenite S-adenosylmethyltransferase	Stress resistance	BacMet
arsA	Arsenical pump-driving	Stress resistance	BacMet
chrE	involved in the processing of chromium-glutathione-complexes	Stress resistance	BacMet
chrF	regulatory protein, involved in Chromate resistance	Stress resistance	BacMet
cnrB	Nickel and cobalt resistance protein	Stress resistance	BacMet
copA	Copper exporting	Stress resistance	BacMet

copB	Copper resistance protein	Stress resistance	BacMet
copZ	Copper chaperone	Stress resistance	BacMet
ctpG	Cadmium transporting	Stress resistance	BacMet
cutO	Multicopper oxidase family protein	Stress resistance	BacMet
czcC	Cobalt-Zinc-Cadmium efflux	Stress resistance	BacMet
czcP	heavy metal translocating P-type ATPase	Stress resistance	BacMet
emrBsm	Putative multidrug resistance protein	Stress resistance	BacMet
mdtG/yceE	Multidrug resistance	Stress resistance	BacMet
merD	Mercury operon coregulator protein	Stress resistance	BacMet
merR	Mercuric resistance operon regulatory protein	Stress resistance	BacMet
merR1	Mercuric resistance operon regulatory protein	Stress resistance	BacMet
nccC	Nickel-cobalt-cadmium resistance protein	Stress resistance	BacMet
ncrB	Metal resistance	Stress resistance	BacMet
pbrA	Pb(II) resistance	Stress resistance	BacMet
pcoA	Copper resistance protein	Stress resistance	BacMet
pcoB	Copper resistance protein	Stress resistance	BacMet
silP	Silver exporting P-type ATPase	Stress resistance	BacMet
tcrB	Copper resistance	Stress resistance	BacMet
terC	Tellurium resistance protein	Stress resistance	BacMet
zntA/yhhO	cadmium, zinc and mercury-transporting ATPase	Stress resistance	BacMet
MGRUST508	Phosphate uptake and utilization	Nutrient cycling	MGRUST
MGRUST533	Heme and Siroheme biosynthesis in plants	Nutrient cycling	MGRUST
MGRUST538	Phosphate uptake	Nutrient cycling	MGRUST
MGRUST569	Iron acquisition	Nutrient cycling	MGRUST
MGRUST571	Iron scavenging cluster	Nutrient cycling	MGRUST
MGRUST747	Nitrogen fixation	Nutrient cycling	MGRUST
MGRUST750	N linked glycosylation	Nutrient cycling	MGRUST
MGRUST831	Phosphoglycerate transport	Nutrient cycling	MGRUST
MGRUST896	P uptake	Nutrient cycling	MGRUST
MGRUST1083	Sulfatases and sulfatase modifying	Nutrient cycling	MGRUST
MGRUST568	Iron acquisition	Nutrient cycling	MGRUST
MGRUST826	Phosphate metabolism	Nutrient cycling	MGRUST

MGRUST1159	Manganese transport	Nutrient cycling	MGRUST
MGRUST540	High affinity phosphate transporter	Nutrient cycling	MGRUST
MGRUST1158	Iron transport	Nutrient cycling	MGRUST
MGRUST1162	Zinc transport	Nutrient cycling	MGRUST
MGRUST1275	Zinc regulatedt	Nutrient cycling	MGRUST
abeS	Quaternary ammonium compound	Nutrient cycling	BacMet
aioX/aoxX	Putative periplasmic phosphite binding	Nutrient cycling	BacMet
bfrA	Iron regulated outer membrane	Nutrient cycling	BacMet
fecD	Fe(3+) dicitrate transport	Nutrient cycling	BacMet
fecE	Fe(3+) dicitrate transport	Nutrient cycling	BacMet
pitA	Low-affinity inorganic phosphate transporter 1	Nutrient cycling	BacMet
pstA	Phosphate transport system permease protein	Nutrient cycling	BacMet
pstB	Phosphate import ATP-binding protein	Nutrient cycling	BacMet
pstC	Phosphate transport system permease protein	Nutrient cycling	BacMet
pstS	Phosphate-binding protein	Nutrient cycling	BacMet
qacG	Quaternary ammonium compound-resistance protein	Nutrient cycling	BacMet
sitA	Iron transporter	Nutrient cycling	BacMet
sugE	Quaternary ammonium compound-resistance protein	Nutrient cycling	BacMet
troC	Zinc transport	Nutrient cycling	BacMet
troD	Zinc transport	Nutrient cycling	BacMet
yfeA	Periplasmic chelated iron-binding	Nutrient cycling	BacMet
yfeB	Chelated iron transport	Nutrient cycling	BacMet
yfeC	Chelated iron transport	Nutrient cycling	BacMet
yfeD	Chelated iron transport	Nutrient cycling	BacMet
zitB/ybgR	Zinc transporter	Nutrient cycling	BacMet
znuB/yebI	High affinity zinc uptake	Nutrient cycling	BacMet
MGRUST720	Mycobacterium virulence operon involved with nitrate dormancy regulon	Pathogenesis	MGRUST
MGRUST1095	T6SS BR toxic	Pathogenesis	MGRUST
MGRUST617	Listeria surface proteins: LPXTG motif	Pathogenesis	MGRUST
MGRUST614	Listeria pathogenicity	Pathogenesis	MGRUST
MGRUST615	Listeria phi A118 like prophages	Pathogenesis	MGRUST

MGRUST712	Mycobacterium virulence operon involved in DNA transcription	Pathogenesis	MGRUST
MGRUST716	Mycobacterium virulence	Pathogenesis	MGRUST
MGRUST717	Mycobacterium virulence operon involved in protein synthesis SSU ribosomal proteins	Pathogenesis	MGRUST
MGRUST721	Mycobacterium virulence operon	Pathogenesis	MGRUST
MGRUST1071	Streptococcus pyogenes Virulome	Pathogenesis	MGRUST
MGRUST1206	Two component Response Regulator of Virulence ResDE	Pathogenesis	MGRUST
MGRUST11	ABC transporter	Microbial metabolism	MGRUST
MGRUST13	ABC transporter dipeptide TC 3 A 1 5 2	Microbial metabolism	MGRUST
MGRUST150	Cytochrome biogenesis	Microbial metabolism	MGRUST
MGRUST791	Glucose catabolism	Microbial metabolism	MGRUST
MGRUST842	Photosystem II type photosynthetic reaction center	Microbial metabolism	MGRUST
MGRUST1112	Terminal cytochrome O ubiquinol oxidase	Microbial metabolism	MGRUST
MGRUST1221	Ubiquinone Menaquinone cytochrome c reductase complexes	Microbial metabolism	MGRUST
MGRUST1231	Unknown carbohydrate utilization cluster Ydj	Microbial metabolism	MGRUST
MGRUST835	Phosphonate metabolism	Microbial metabolism	MGRUST
MGRUST462	Fe S cluster assembly	Microbial metabolism	MGRUST
arrB	4Fe-4S ferredoxin	Microbial metabolism	BacMet
modA	Molybdenum ABC transporter, periplasmic molybdenum-binding protein	Microbial metabolism	BacMet
modB	Molybdenum ABC transporter, permease protein	Microbial metabolism	BacMet
vcaM	ABC transporter	Microbial metabolism	BacMet

Supplmentary References

1. Byrnes, J.E.K., et al., Investigating the relationship between biodiversity and ecosystem multifunctionality: challenges and solutions. *Methods Ecol. Evol.* 5, 111-124 (2014).
2. Rillig, M.C., et al., The role of multiple global change factors in driving soil functions and microbial biodiversity. *Science* 366, 886-890 (2019).
3. Tóth, G., Hermann, T., Da Silva, M. R. & Montanarella, L. Heavy metals in agricultural soils of the European Union with implications for food safety. *Environ. Int.* 88, 299-309 (2016).
