# Supplementary material for: Soil contamination in nearby natural areas mirrors that in urban greenspaces worldwide
Source: Nat Commun. 2023 Mar 27;14:1706. doi: 10.1038/s41467-023-37428-6 (PMC10042830; doi:10.1038/s41467-023-37428-6)
Supplement: Supplementary file 3 — Reporting Summary [file 41467_2023_37428_MOESM3_ESM.pdf]

nature portfolio

Corresponding author(s): Yu-Rong Liu(yrliu@mail.hzau.edu.cn)
Manuel Delgado-Baquerizo
( m.delgadobaquerizo@gmail.com)

Last updated by author(s): Feb 24, 2023



# Reporting Summary

Nature Portfolio wishes to improve the reproducibility of the work that we publish. This form provides structure for consistency and transparency in reporting. For further information on Nature Portfolio policies, see our Editorial Policies and the Editorial Policy Checklist.

## Statistics

For all statistical analyses, confirm that the following items are present in the figure legend, table legend, main text, or Methods section.

| n/a | Confirmed | |
|---|---|---|
| ☐ | ☒ | The exact sample size (*n*) for each experimental group/condition, given as a discrete number and unit of measurement |
| ☐ | ☒ | A statement on whether measurements were taken from distinct samples or whether the same sample was measured repeatedly |
| ☐ | ☒ | The statistical test(s) used AND whether they are one- or two-sided<br>*Only common tests should be described solely by name; describe more complex techniques in the Methods section.* |
| ☐ | ☒ | A description of all covariates tested |
| ☐ | ☐ | A description of any assumptions or corrections, such as tests of normality and adjustment for multiple comparisons |
| ☐ | ☒ | A full description of the statistical parameters including central tendency (e.g. means) or other basic estimates (e.g. regression coefficient) AND variation (e.g. standard deviation) or associated estimates of uncertainty (e.g. confidence intervals) |
| ☐ | ☒ | For null hypothesis testing, the test statistic (e.g. *F*, *t*, *r*) with confidence intervals, effect sizes, degrees of freedom and *P* value noted<br>*Give P values as exact values whenever suitable.* |
| ☒ | ☐ | For Bayesian analysis, information on the choice of priors and Markov chain Monte Carlo settings |
| ☒ | ☐ | For hierarchical and complex designs, identification of the appropriate level for tests and full reporting of outcomes |
| ☐ | ☒ | Estimates of effect sizes (e.g. Cohen's *d*, Pearson's *r*), indicating how they were calculated |

*Our web collection on statistics for biologists contains articles on many of the points above.*

## Software and code

Policy information about availability of computer code

| | |
|---|---|
| Data collection | Data for this manuscript are from lab analyses of paired urban greenspaces and adjacent natural areas of 56 municipalities in 17 countries across six continents, and  Climatic variables were collected from the Worldclim database (https://www.worldclim.org) and socio-economic parameters are from city develop reports, using ArcGIS V10.6 software. Soil properties and contaminants were measured as explained in the Method section of our manuscript. |
| Data analysis | The SeqPrep 1.2 software (https://github.com/jstjohn/SeqPrep) was used to remove adapter sequences. Then, the library sickle (https://github.com/najoshi/sickle) was used to trim the reads from the 5' end to 3'end using a sliding window. The differences in contents of contaminants from paired urban greenspaces and natural areas were performed using nested PERMANOVA  in the R package "Vegan" ("adonis" routine) in R 4.0.3. |

For manuscripts utilizing custom algorithms or software that are central to the research but not yet described in published literature, software must be made available to editors and reviewers. We strongly encourage code deposition in a community repository (e.g. GitHub). See the Nature Portfolio guidelines for submitting code & software for further information.

## Data

Policy information about availability of data

All manuscripts must include a data availability statement. This statement should provide the following information, where applicable:

- Accession codes, unique identifiers, or web links for publicly available datasets
- A description of any restrictions on data availability
- For clinical datasets or third party data, please ensure that the statement adheres to our policy

The data that support the findings of this study publicly available at figshare: https://figshare.com/articles/dataset/DATASET_Yu-RongLiu_20230216/22107971; DOI: 10.6084/m9.figshare.22107971;
All code associated with our analyses in this study is available at https://figshare.com/articles/dataset/Rscript_Yu-RongLiu_20230216/22107818; https://doi.org/10.6084/m9.figshare.22107818.

## Human research participants

Policy information about studies involving human research participants and Sex and Gender in Research.

| | |
|---|---|
| Reporting on sex and gender | N/A |
| Population characteristics | N/A |
| Recruitment | N/A |
| Ethics oversight | N/A |

Note that full information on the approval of the study protocol must also be provided in the manuscript.

# Field-specific reporting

Please select the one below that is the best fit for your research. If you are not sure, read the appropriate sections before making your selection.

☐ Life sciences ☐ Behavioural & social sciences ☒ Ecological, evolutionary & environmental sciences

For a reference copy of the document with all sections, see nature.com/documents/nr-reporting-summary-flat.pdf

# Ecological, evolutionary & environmental sciences study design

All studies must disclose on these points even when the disclosure is negative.

| | |
|---|---|
| Study description | This study is based on a standardized field survey includes paired urban greenspaces and adjacent natural areas of 56 locations from 17 countries across six continents, where we collected composite topsoil samples. The selected sites provide a large representation of soil contaminants in the areas. Based on this survey, we aim to compare levels of multiple soil contaminants in urban greenspaces and adjacent natural areas, and examine the potential influence of soil contaminants on functional microbial traits. |
| Research sample | We collected surface soils (top 5 cm) from the 112 ecosystems, and obtained 336 composite soil samples in total. Due to sample availability and resource limitation, we did not analyze all soils for each category of contaminant. Many of the analyses included in this study (e.g., polymer identification of microplastics and 46 pesticide residues) are highly costly and time-consuming, which require a relatively large amount of sample for contaminant extraction (see Methods in the text).We thus focused on the subset of sites potentially impacted by specific contaminants according to advice from local greenspace managers and environmental scientists. Therefore, we attempted to cover the entire gradient of conditions by focusing on a subset of the samples. Thus, we measured (metal(loid)s and ARGs in all the 336 composite samples from 112 plots, microplastics in 64 composite samples from the selected 64 plots and pesticide residues in 54 composite samples from the selected 54 plots. In all cases, the subsets of samples were selected to cover the entire biogeographic range along with a broad range of environmental gradients. |
| Sampling strategy | A 30 m × 30 m plot (900 m2) consisting of three parallel transects of equal length was surveyed at each location . These plots were selected to represent the most common environments within ecosystems. We then collected surface soils (top 5 cm) from the 112 ecosystems, and obtained 336 composite soil samples in total. To account for spatial heterogeneity, we collected three sampling points for a composite sample under the most common environments found at each site. Our samples were selected to cover the entire biogeographic range along with a broad range of environmental gradients worldwide (Supplementary Fig. 1 and Table 1) |
| Data collection | Soil pH was determined on a 1: 5 soil/water extract using a pH electrode. Total carbon and nitrogen in the soil were analyzed using an elemental analyzer. Soil organic carbon was measured using the same elemental analyzer after fumigation with HCl . Total phosphorus was determined using an inductively coupled plasma optical emission spectrometer after digestion using nitric-perchloric acid. For the analyses of metal(loid)s, soil was digested by a MARS microwave digestion system using mixed acids. The pesticide residues in soils were analyzed by high-performance liquid chromatography coupled to a triple quadrupole tandem mass spectrometer (HPLC-MS/MS). Microplastics in soil were pretreated using Fenton's oxidization method, and analyzed and counted |

visually using a binocular microscope glass. A high-throughput quantitative PCR (HT-qPCR) based chip was used to quantify ARGs on the Wafergen SmartChip Real-Time PCR System. Functional genes were analysed through shotgun sequencing.

| | |
|---|---|
| Timing and spatial scale | Sample collection of soils of 56 municipalities from 17 countries across six continents took place between 2017 and 2019. |
| Data exclusions | No data were excluded in the analyses. |
| Reproducibility | Within the manuscript, we clearly state all the steps taken to ensure the reproducibility of the study. We include descriptions of standard sampling and analytical protocols and the identification of all code packages used. |
| Randomization | N/A |
| Blinding | N/A |

Did the study involve field work? ☒ Yes ☐ No

## Field work, collection and transport

| | |
|---|---|
| Field conditions | Field sampling was conducting a wide range of environmental conditions, and the mean annual precipitation and temperature. ranged from 210 to 1577 mm and 3.1 ºC to 26.4 ºC, respectively. More details can be found in Methods section of the manuscript. |
| Location | Detailed information on geographical coordinates of sampling locations included in this study were presented in Supplementary Figure 1 and Supplementary Table 1. |
| Access & import/export | All soil samples were collected by our co-authors from global institutes under permits of national and local governments. |
| Disturbance | This study did not cause any environmental disturbance. |

# Reporting for specific materials, systems and methods

We require information from authors about some types of materials, experimental systems and methods used in many studies. Here, indicate whether each material, system or method listed is relevant to your study. If you are not sure if a list item applies to your research, read the appropriate section before selecting a response.

### Materials & experimental systems

| n/a | Involved in the study |
|---|---|
| ☒ | ☐ Antibodies |
| ☒ | ☐ Eukaryotic cell lines |
| ☒ | ☐ Palaeontology and archaeology |
| ☒ | ☐ Animals and other organisms |
| ☒ | ☐ Clinical data |
| ☒ | ☐ Dual use research of concern |

### Methods

| n/a | Involved in the study |
|---|---|
| ☒ | ☐ ChIP-seq |
| ☒ | ☐ Flow cytometry |
| ☒ | ☐ MRI-based neuroimaging |
